# Narrowband, Angle-Tunable, Helicity-Dependent Terahertz Emission from Nanowires of the Topological Dirac Semimetal Cd_3_As_2_

**DOI:** 10.1021/acsphotonics.3c00068

**Published:** 2023-04-03

**Authors:** Jessica L. Boland, Djamshid A. Damry, Chelsea Q. Xia, Piet Schönherr, Dharmalingam Prabhakaran, Laura M. Herz, Thorsten Hesjedal, Michael B. Johnston

**Affiliations:** † Photon Science Institute, Department of Electrical and Electronic Engineering, University of Manchester, Manchester M13 9PL, U.K.; ‡ Department of Physics, University of Oxford, Clarendon Laboratory, Parks Road, Oxford OX1 3PU, U.K.

**Keywords:** terahertz, photonics, nanowires, Dirac semi-metal, helicity-dependent emission

## Abstract

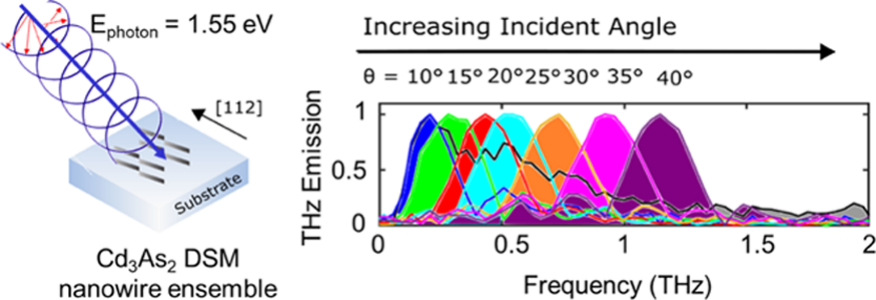

All-optical control of terahertz pulses is essential for the development of optoelectronic devices for next-generation quantum technologies. Despite substantial research in THz generation methods, polarization control remains difficult. Here, we demonstrate that by exploiting band structure topology, both helicity-dependent and helicity-independent THz emission can be generated from nanowires of the topological Dirac semimetal Cd_3_As_2_. We show that narrowband THz pulses can be generated at oblique incidence by driving the system with optical (1.55 eV) pulses with circular polarization. Varying the incident angle also provides control of the peak emission frequency, with peak frequencies spanning 0.21–1.40 THz as the angle is tuned from 15 to 45°. We therefore present Cd_3_As_2_ nanowires as a promising novel material platform for controllable terahertz emission.

## Introduction

The generation and control of terahertz (THz) pulses and their associated ultrafast photoinduced currents is essential for the development of coherent optoelectronic quantum devices and fundamental materials research. High-energy, few-cycle THz pulses play a crucial role in broadband time-resolved^1,2^ and nonlinear^3,4^ spectroscopy, polarimetry,^5^ ultrafast time-resolved microscopy,^6,7^ electron acceleration,^8,9^ and 6G communication.^10,11^ Multi-cycle, narrowband THz emission has applications in imaging^12,13^ and security,^14,15^ and as pumps for selective excitation and control of light–matter interactions,^16^ including topology switching,^17,18^ field-induced ferroelectricity,^19^ and the Higgs mode in superconductors.^20^ These applications call for a THz source that provides not only both broadband and narrowband operations for time-resolved and frequency-resolved experiments, respectively, but also high-power, tunable peak emission frequency and polarization control.

There has been a wealth of research into THz generation methods, namely, difference-frequency generation (DFG) with multi-color laser pulses^21,22^ and optical rectification (OR) of a single broadband optical pulse in nonlinear crystals.^2,23^ A range of materials, including semiconductors,^24,25^ organic crystals,^26^ and ferromagnets,^27^ have been examined with demonstration of both high-energy single-cycle and multi-cycle THz pulses. For DFG, the central emission frequency and bandwidth rely on the difference between the wavelengths of the optical pulses used. For OR generation processes, they depend on the pulse duration of the optical pulse used as well as material properties, such as phase matching and phonon resonances. Polarization control usually requires pulse shaping with two-color filamentation in an air plasma, yet spintronic emitters have also recently demonstrated polarization control. In these emitters, the polarization of the emitted THz radiation can be tuned by tailoring the magnetic field profile applied to the anti-ferromagnetic/ferromagnetic heterostructure.^28−30^ Alongside this, the exploitation of band structure topology in Dirac systems, such as topological insulators (TIs), Weyl semi-metals (WSMs), and Dirac semi-metals (DSMs), has also emerged as an alternative approach for polarization control across the THz regime.^31,32^

Dirac materials are characterized by Weyl nodes, where the conduction and valence bands touch at a discrete point in momentum space, and host topologically protected massless Dirac fermions. In TIs, these points occur at the surface, producing topological conducting surface states, while the bulk retains the insulating (or, in practice, semiconducting) behavior.^33^ WSMs and DSMs exhibit a linear, gapless energy-momentum dispersion in all three dimensions: with two opposite-chirality nodes for WSMs^34^ and two degenerate Weyl nodes for DSMs.^35^ These materials have demonstrated several exotic, optoelectronic phenomena, such as quantized photocurrents,^36,37^ photo-induced anomalous Hall and quantum Hall effects,^38^ spin-polarized photocurrents,^39,40^ giant magnetoresistance,^41,42^ and Floquet–Bloch states.^43^ Many of their distinctive features—linear band dispersion and Berry curvature—are closely related to their response to light, which has been utilized within devices, including broadband photodetectors^44^ and THz modulators.^45^ However, they have another significant advantage: they host helicity-dependent photocurrents due to spin-momentum locking. A photon with definite helicity can induce a transition that flips the direction of spin via the circular photogalvanic effect (CPGE) to create an electron–hole pair that will drive a current. This helicity-dependent photocurrent is directly proportional to the Berry curvature, providing a direct experimental measure of the quantized topological charge at the Weyl nodes.^36,46,47^

As a result, there has been renewed interest in photo-induced currents and subsequent THz emission from topological materials. Such experiments may not only provide insights into their topological non-trivial behavior but also demonstrate a material platform for achieving all-optical control in THz optoelectronic devices. Extensive studies have been carried out on TIs and WSMs. For example, nonlinear THz emission has been observed in Bi_2_Te_3_ thin film TIs, where contributions from nonlinear surface photocurrents surpass drift and diffusion currents.^48^ Helicity-dependent photocurrents have also been observed in Sb_2_Te_3_ thin film TIs with direct control of the polarity of the corresponding emitted THz radiation through selection of an incident angle and polarization of the optical pulse excitation.^49^ In the WSM TaAs, helicity-dependent photocurrents via CPGE and shift currents owing to the linear photogalvanic effect (LPGE) have both been observed.^50,51^ Recent investigations into the photon-energy dependence of THz emission in the chiral WSM have also provided evidence that chiral Weyl fermions are responsible for the generation of ultrafast quantized currents via CPGE at high photon energies.^52^

DSMs are particularly promising for achieving polarization control, as they have been shown to exhibit strong optical nonlinearities^53^—a pre-requisite for the generation of high-energy pulses. It has also been predicted that they can be switched into a WSM via breaking time-reversal or inversion symmetry, providing a platform for helicity-dependent photocurrents.^43^ In particular, cadmium arsenide (Cd_3_As_2_) has emerged as a 3D analogue of graphene,^54^ offering ultra-high electron mobilities and Fermi velocities observed in its 2D counterpart, yet with the advantage of large-area fabrication, stability, and broadband absorption properties inherent to its 3D nature. Interest is now extended to the THz range, where its nonlinearity has been probed through high-harmonic generation via THz-pump THz-probe spectroscopy,^55,56^ and intense THz pulses have generated Raman phonon coherences that can drive topological phase transitions.^18^ Alongside these, theoretical studies have predicted an enhancement in optical-to-terahertz conversion efficiency in Cd_3_As_2_ that is 5000 times higher than the current state of the art for high-energy narrowband THz emission—lithium niobate.^57^ However, this enhancement has yet to be realized experimentally.

In this letter, we present helicity-dependent THz emission in the DSM Cd_3_As_2._ By performing THz emission spectroscopy on a bulk Cd_3_As_2_ single crystal and nanowire ensemble, we show that both few-cycle and narrowband multi-cycle THz pulses can be generated upon near-infrared photoexcitation by switching the polarization of light from linear to circular polarization, respectively. For both the bulk crystal and nanowire ensemble, we independently extract the time-domain traces of coefficients describing the emission mechanisms—CPGE, LPGE, photon drag effects (PDE), OR, and bulk thermal effects. At normal incidence, no CPGE occurs and the dominant emission mechanism is OR and bulk thermal effects (including PDE), producing THz pulses with spectral extension out to ∼1 THz. At oblique incidence, helicity-dependent THz pulses are instead generated predominantly via CPGE, as inversion symmetry is broken within the single crystal and the reduction in symmetry in the nanowire ensemble switches the DSM into a WSM.^58−61^ These THz pulses have a narrow bandwidth of ∼0.4 THz, and their emission frequency can be tuned between 0.23 and 1.40 THz by varying the incident angle from 10 to 45°, rendering Cd_3_As_2_ a promising candidate as a frequency-tunable THz pump for frequency-resolved experiments and an all-optical source for coherent quantum optoelectronic devices.

## Results and Discussion

### Azimuthal Dependence of THz Emission in Cd_3_As_2_ at Normal Incidence

**Figure 1:**
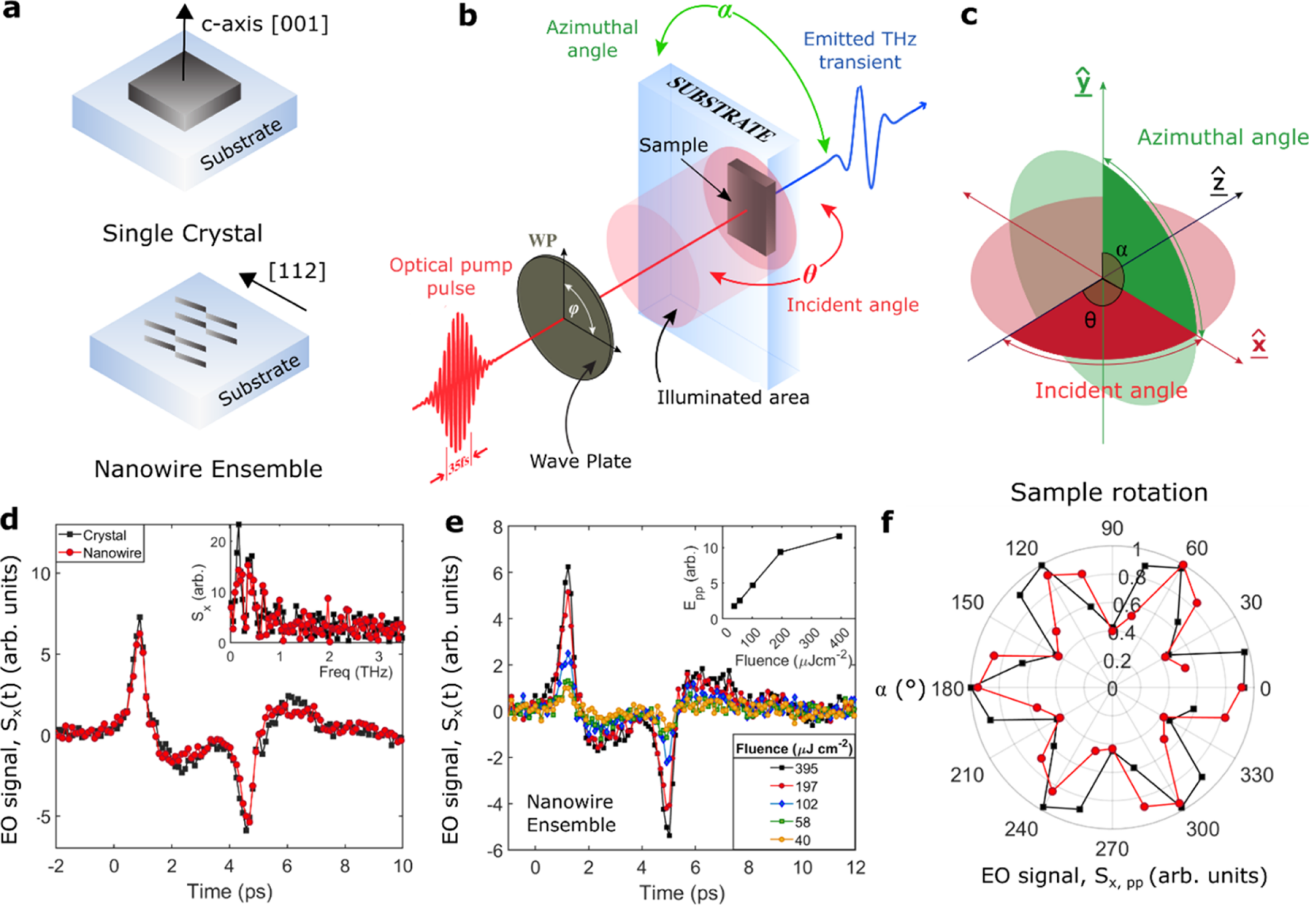
Schematic diagram depicting (a) the sample geometry; (b) the experimental setup; and (c) the plane of the sample azimuthal angle, α, and incident angle, θ, of the optical pump. An optical pump pulse (800 nm, 35 fs) passes through a quarter wave plate with polarization angle, φ, and photoexcites either a bulk Cd_3_As_2_ single crystal or an aligned nanowire ensemble at incident angle, θ. The emitted THz radiation is focused onto a (110) ZnTe crystal for electro-optic sampling to detect the emission polarized in the *x*-direction. (d) The electro-optic signal of the emitted THz waveforms for the bulk single crystal (black squares) and nanowire ensemble (red circles) recorded at α = 180° for optical photoexcitation at a fluence of 395 μJ cm^–2^. Inset: FFT spectra of the THz waveforms. (e) Fluence dependence of THz waveforms for the nanowire ensemble at α = 180°. Inset: peak-to-peak value of the THz amplitude as a function of photoexcitation fluence. (f) Polar plot of the peak-to-peak values of the emitted THz amplitude as a function of the azimuthal angle under illumination with linearly polarized NIR photons at normal incidence for the crystal (black squares) and nanowire ensemble (red circles).

To investigate photoinduced currents in Cd_3_As_2_, a standard THz emission spectroscopy system in transmission configuration was used to examine THz pulses generated in both a bulk Cd_3_As_2_ crystal and a nanowire ensemble (see Methods and Figure S3 in the Supporting Information). The bulk single Cd_3_As_2_ crystal was grown inside a two-zone furnace from stoichiometric amounts of Cd and As elements.^62^ It is approximately 1 mm^3^ in size and has a tetragonal crystal structure belonging to the centrosymmetric *I*4_1_/*acd* space group. The Cd_3_As_2_ nanowire ensembles were grown via a self-catalyzed vapor–solid growth process,^61^ and they crystallized in the non-centrosymmetric space group *I*4_1_*cd*. The nanowire ensemble contained a distribution of nanowires with an average diameter of 100 nm and an average length of 15 μm. Both samples were supported on a 2 mm-thick z-cut quartz substrate, which exhibits low dispersion and is transparent in the THz range.^63^ For the nanowire ensemble, the nanowires were transferred to the quartz substrate by preferentially rubbing in one direction to ensure that their nanowire axes were predominantly aligned in one direction (see Figure S1 in the Supporting Information) to avoid any artifacts due to the nanowire geometry itself and enable control via the inherent nanowire polarization anisotropy.^64^ The crystal structure and the sample geometry for both the bulk crystal and the nanowire ensemble are shown in Figure 1a.

For the centrosymmetric bulk crystal, the band structure resembles that of a topological DSM with two symmetry-protected doubly-degenerate Dirac cones along the [001] direction in the bulk and the [112] direction on the surface. The non-centrosymmetric nanowire ensemble also possesses fourfold degenerate Dirac points; however, the lack of inversion symmetry splits the in-plane band dispersions away from the Dirac point.^37^Figure 1b,c shows schematic diagrams of the experimental setup. Linearly polarized optical NIR “pump” pulses are passed through a quarter wave plate at an angle, φ, to set the desired polarization, before being loosely focused on the sample with a full width at half-maximum (FWHM) of 8.43 mm at an incident angle, θ. The optical pump pulse photoexcites electrons into higher energy states across the Dirac cone, creating non-Dirac electron–hole pairs. Some of these charge carriers then relax back into the Dirac cone or into bulk bands on picosecond timescales, inducing ultrafast photocurrents that generate THz radiation. These emitted THz pulses are focused on a (110) ZnTe nonlinear crystal, where their amplitude and phase are extracted via free-space electro-optic sampling. We note that the THz emission was measured over a temporal window that excluded any potential reflections from the substrate and was detected in the horizontal direction, so it displays only the emission components in the *x*-direction. Figure 1d shows the time-domain electric field transients of the THz pulses generated from both the single crystal and the nanowire ensemble with the Fourier transform shown in the inset. For both samples, the emitted amplitude was found to be an order of magnitude smaller than a 1 mm ZnTe crystal (see Figure S13 in the Supporting Information) yet displayed emission with a spectral extension out to ∼1 THz. Interestingly, the THz emission amplitude for both the single crystal and nanowire ensemble is comparable, despite the reduction in size associated with the nanowire geometry, whose average diameter of 100 nm is 4 orders of magnitude smaller than the 1 mm thick single crystal. This highlights the promise of using Cd_3_As_2_ nanowires for future single-nanowire high-power THz emitters or detectors.

We propose the following potential mechanisms for the THz emission observed at normal incidence for the case of photoexcitation with 1.55 eV pulses into high-energy bulk conduction bands: shift currents via LPGE, injection currents via CPGE, OR, PDE, and bulk thermoelectric current related to the linear absorption in Cd_3_As_2_.^65^ All of these mechanisms depend monotonically on the power of the optical pump pulse: OR, CPGE, and LPGE are resonant second-order nonlinear processes and quadratic with electric field, whereas PDE and thermoelectric currents are bulk processes that depend linearly on the absorption. Figure 1e displays the emitted THz waveforms for photoexcitation fluences ranging from 40 to 395 μJ cm^–2^. The absolute peak-to-peak value of the THz emission amplitude increases linearly with increasing pump fluence (inset) up until 200 μJ cm^–2^, where it begins to saturate. This saturation at high photoexcitation fluences is attributed to the saturation of interband transitions and low-frequency screening of the response, reminiscent of the Moss–Burstein effect observed in semiconductors.^66^ However, the observed fluence dependence confirms the linear dependence of the THz emission amplitude on the optical pump power.

Of these processes, OR and PDE are dependent on crystal orientation. OR depends on the effective second-order nonlinear susceptibility, which is described by a third-rank tensor. The effective second-order nonlinear susceptibility consists of both the second-order nonlinear susceptibility, χ_2_, and a DC-field-induced effect, which is allowed for above-bandgap excitation via the third-order nonlinear susceptibility, χ_3_ and a surface field. In centrosymmetric crystals (i.e., our Cd_3_As_2_ bulk crystal), χ_2_ vanishes in the bulk, but takes on a non-zero value at the surface, where the inversion symmetry is broken, providing a route for surface OR. The PDE is a bulk effect associated with the linear momentum transfer between incident photons and electrons and is described by a fourth-rank tensor (see Section S5 in the Supporting Information). Both should display an azimuthal dependence following the crystal symmetry. To therefore determine the effect of crystal symmetry on the emitted THz radiation, both samples were rotated about an azimuthal angle, α. As shown in Figure 1a, the centrosymmetric bulk crystal was orientated with the [001] *c*-axis parallel to the wavevector of light at normal incidence (i.e., along *ẑ* in Figure 1c for θ = 0°), so that the pump pulse was incident on the (112) surface. Similarly, the non-centrosymmetric nanowire ensemble was aligned with the nanowire axis and thereby with the (112) surface parallel to the horizontal linear polarization of the optical pump beam at φ = 0° (i.e., along *x̂* in Figure 1c for α = 0°). Figure 1f depicts the crystal-orientation (or azimuthal) dependence of the absolute peak-to-peak value of the emitted THz radiation for photoexcitation with linearly polarized NIR photons (φ = 0°) at normal incidence (θ = 0°). Both the centrosymmetric single crystal and non-centrosymmetric nanowire ensemble display a sixfold symmetry, with maxima at α = 0, 60, and 120°. The observed sixfold symmetry is in contrast to the expected *C*_4_ symmetry of the bulk yet matches the *C*_3_ symmetry of the (112) surface more closely.^67^ Previous ARPES measurements have demonstrated the presence of two bulk bands and a surface band that have a Dirac cone on the (112) surface.^67^ This experimental observation suggests that photocurrents from both bands contribute to the THz emission, with a dominant contribution from photocurrents at the surface.

### Polarization Dependence of THz Emission at Normal Incidence

To further elucidate the mechanisms behind the THz emission observed at normal incidence, we observe the polarization dependence of the THz signals. The degree of circular polarization of the incident light was controlled by rotating the quarter wave plate by an angle φ and the emitted THz electro-optic signal was measured as a function of time delay at two different sample orientations: α = 180 and 90°, which correspond to a maximum and minimum in THz emission, respectively (Figure 1f). For α = 180°, we expect rectification and photon drag currents to dominate as dictated by the observed azimuthal dependence and crystal symmetry; whereas at α = 90°, these effects should be minimized.

**Figure 2:**
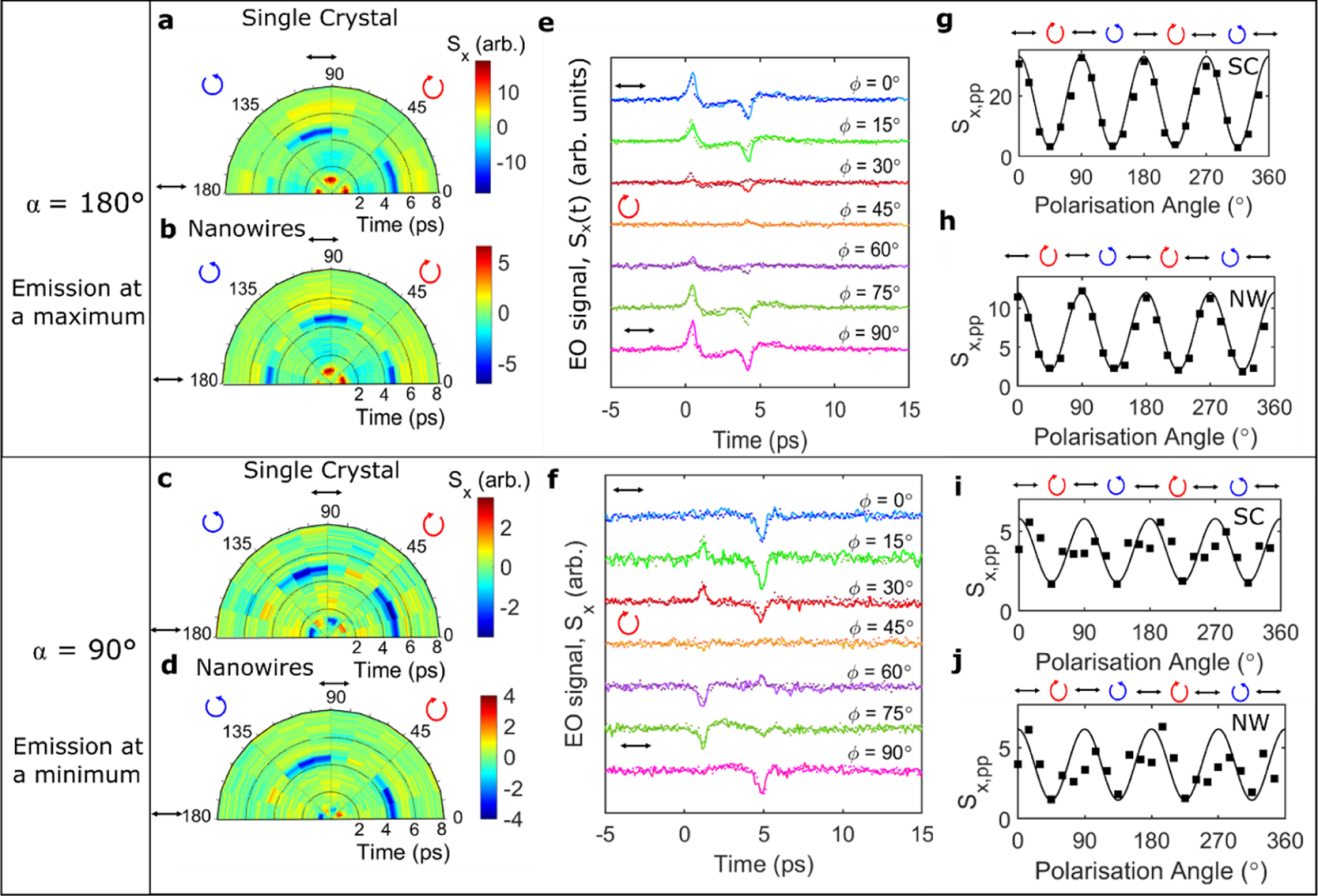
(a–d) Polar plots of THz waveforms as a function of polarization angle, φ, at θ = 0° for the bulk crystal and nanowire ensemble, respectively, taken at (a,b) α = 180° (when THz emission is at a maximum in Figure 1f) and (c,d) α = 90° (when THz emission is minimized in Figure 1f). The time delay is plotted along the radius and the polarization angle against the circumference. The color bar represents the amplitude of the THz emission. (e,f) THz waveforms recorded at θ = 0° and various polarization angles for the bulk crystal (solid lines) and nanowire ensemble (dotted lines) for crystal orientations at (e) α = 180° and (f) α = 90°. (g–j) Peak-to-peak value of the THz amplitude as a function of polarization angle, φ, at θ = 0° for the crystal and nanowire ensemble, respectively, at (g,h) α = 180° and (i,j) α = 90°. The symbols indicate the experimental data, and the solid line indicates the fitting result from eq 1.

Figure 2a–d shows polar color plots of the THz waveforms as a function of polarization angle, φ. Exemplar time-domain traces of the emitted THz radiation are plotted for specific polarization angles in Figure 2e,f, for both the centrosymmetric bulk crystal (solid lines) and the non-centrosymmetric nanowire ensemble (dashed lines). On inspection of the time-domain traces for different azimuthal angles, we notice a change in the THz waveform. For α = 180°, two clear peaks (at time delays of 0.9 and 4.7 ps) are observed in the waveform, with the first peak a maximum. We note that these two peaks are not necessarily desirable for spectroscopic applications, as they produce a strong modulation in the emitted frequency spectrum. However, the azimuthal angle of the crystal and the nanowire ensemble could be tuned to produce a more suitable temporal profile. For example, at α = 90°, the magnitude of the first peak (*t* = 0.9 ps) in the THz waveform is significantly reduced, whereas the second peak (*t* = 4.7 ps) becomes more prominent. This suggests that the two peaks could be caused by separate emission mechanisms. A similar observation was previously made for THz generation in the topological surface states and bulk bands in TIs.^68^ Alternatively, the two peaks could be due to photocurrent processes with different relaxation times. In particular, Cd_3_As_2_ thin films have been shown to have relaxation times on the order of ∼6 ps,^69^ which is close to the observed time delay (3.8 ps) between the two peaks. Cd_3_As_2_ also exhibits a strong electron–hole asymmetry,^59^ so electrons and holes are likely to relax into the bulk and surface bands with different relaxation times. This could lead to a time delay and polarity change between the emission from electron and hole currents, producing the two peaks observed in our experiments.

However, at both azimuthal angles, the samples show a clear polarization dependence: the THz emission is maximized following photoexcitation with linearly polarized NIR photons yet negligible when excited with circularly polarized light. The experimental data were fitted with the following equation^49,50,70,71^
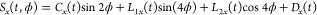
1where *C*_*x*_(*t*) defines the contribution from helicity-dependent currents, *L*_1*x*_(*t*) and *L*_2*x*_(*t*) result from helicity-independent photocurrents, and *D*_*x*_(*t*) represents polarization-independent currents. *C*_*x*_(*t*) can contain contributions from both CPGE and the circular photon drag effect (CPDE). *L*_1*x*_(*t*) depends on the linear polarization of light and is the quadratic nonlinear response owing to LPGE. *L*_2*x*_(*t*) and *D*_*x*_(*t*) are thermal effects associated with linear absorption, with *L*_2*x*_(*t*) describing currents arising from PDE and *D*_*x*_(*t*) depicting the contribution from rectification and bulk photovoltaic currents.^47^Figure 2g–j shows the absolute peak-to-peak value of the emitted THz amplitude (squares) against the polarization angle, φ, alongside the fits from eq 1 (solid lines). The equation fits the experimental data well and clearly shows a cos 4ϕ periodicity, suggesting that the observed THz radiation arises predominantly from helicity-independent photocurrents at normal incidence.

**Figure 3:**
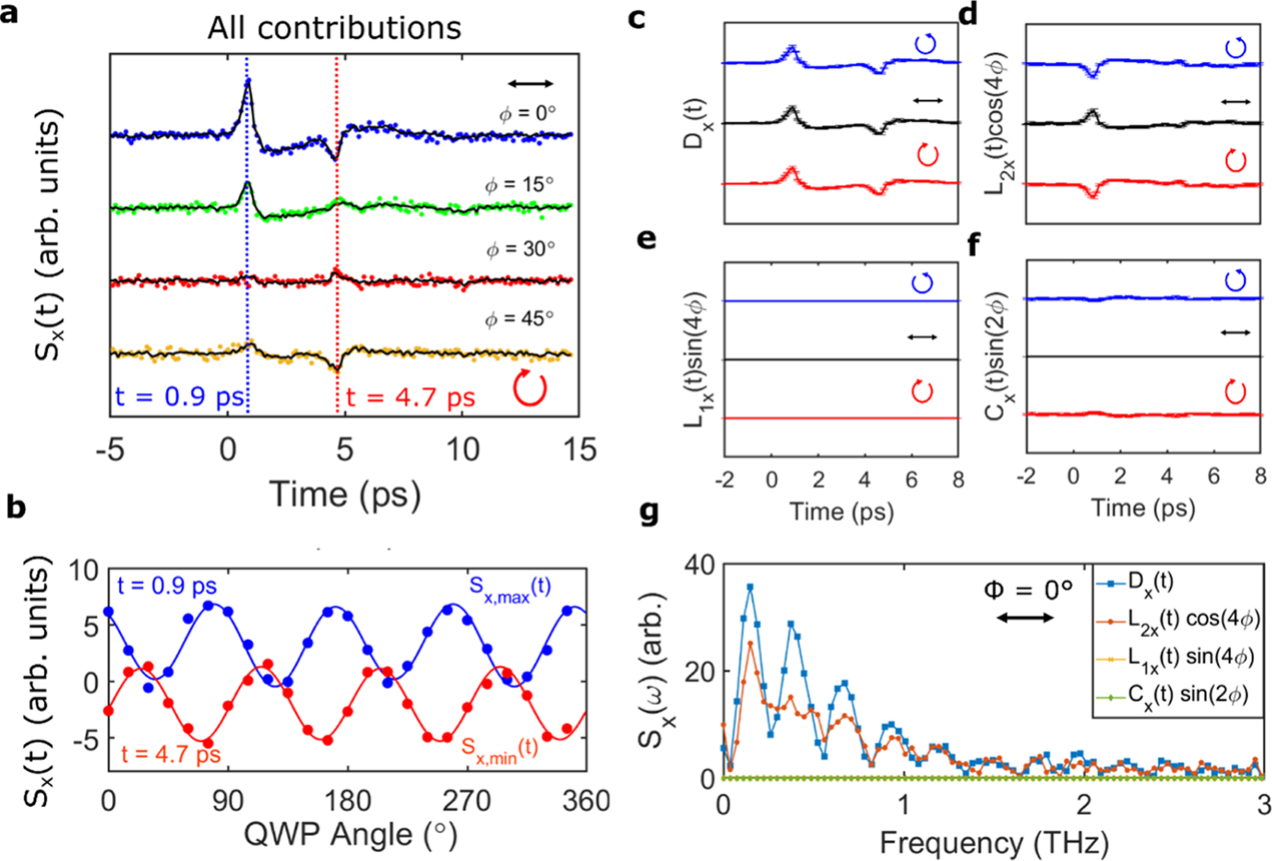
(a) Emitted THz waveforms for the nanowire ensemble under illumination from linearly (φ = 0°), elliptically (φ = 15, 30°), and circularly polarized (φ = 45°) NIR photons at normal incidence. The symbols indicate the experimental data, and the solid lines indicate the fitting result from eq 1. (b–e) Time-domain traces of the contributions to the electro-optic signal, *S*_*x*_(*t*) from the terms *C*_*x*_(*t*) sin(2φ), *L*_1*x*_(*t*) sin(4φ), *L*_2*x*_(*t*) cos(4φ), and *D*(*t*) extracted from eq 1 for photoexcitation with right-handed circularly (blue), linearly (black), and left-handed circularly polarized (red) NIR photons (i.e., φ = −45, 0, +45°). The y-axes are all the same scale, and the extracted contributions are plotted with the 95% confidence intervals calculated from the fitting process. (f) THz amplitude as a function of polarization angle, φ, at a time delay corresponding to dashed lines in (a), *t* = 0.9 ps (blue) and *t* = 4.7 ps (red). (g) Corresponding spectra for *C*_*x*_(*t*) sin(2φ) (green diamonds), *L*_1*x*_(*t*) sin(4φ) (yellow stars), *L*_2*x*_(*t*) cos(4φ) (red circles), and *D*(*t*) (blue squares) obtained by FFT of extracted coefficient amplitudes in (b–e) for illumination with linearly polarized NIR photons at normal incidence.

We further confirm this hypothesis by extracting the time-domain traces of all the fitted coefficients in eq 1 (solid lines). By fitting the polarization angle dependence of the emitted signal for each time delay, the full waveform arising from all contributions could be obtained. The calculated electric-field waveform matched extremely well with the experimental data, as shown by the solid lines in Figure 3a for the nanowire ensemble at α = 180°. Examples of the fitted φ-dependence are also shown in Figure 3b for two different time delays: *t* = 0.9 and 4.7 ps, corresponding, respectively, to the first peak and second peak in the waveform. Again, the fits show good agreement with the experimental data, clearly following the same cos 4ϕ periodicity. From these fits, the time-dependent coefficients were then plotted independently to isolate the photocurrent contributions. Figure 3c–f shows these time-domain traces for the nanowire ensemble for right-handed circular (blue), horizontal-linear (black), and left-handed circular (red) polarization.

The shape of the THz waveform is replicated by the addition of the *D*_*x*_(*t*) and *L*_2*x*_(*t*) coefficients, with the polarization-independent *D*_*x*_(*t*) dominating at α = 180° and *L*_2*x*_(*t*) for α = 90°. This hints that the origin of these photocurrents is a combination of rectification and bulk thermoelectric currents. The *L*_2*x*_(*t*) contribution also follows the same cos 4ϕ periodicity as the absorption within these materials. The non-centrosymmetric nanowire ensemble has a strong polarization-sensitive anisotropy in optical absorption. Illumination with NIR photons linearly polarized parallel (perpendicular) to the nanowire axis produces a maximum (minimum) in absorption.^64^ The nanowire geometry therefore provides a means to control their absorption and subsequent THz emission, by using a half wave plate to rotate the degree of polarization between horizontal (parallel to the nanowire axis) and vertical (perpendicular to the nanowire axis). A maximum and minimum in the emitted THz signal were observed for the nanowire ensemble for horizontal and vertical polarization, respectively, confirming that *L*_2*x*_(*t*) is related to linear absorption (see Section S5 in the Supporting Information) and therefore is related to bulk PDE. The recorded electro-optic signal also changes polarity with linearly and circularly polarized light, as expected for the PDE (see Section S6 in the Supporting Information).

On the other hand, *C*_*x*_(*t*) and *L*_1*x*_(*t*) both appear to have a negligible contribution. *L*_1*x*_(*t*) describes shift currents, which arise following interband optical excitation due to a spatial shift in the charge carrier position that generates a time-dependent dipole moment. These currents can only be generated by linearly polarized light and are related to the nonlinear optical conductivity, σ_*ijk*_^(2)^,^72^ which imposes symmetry constraints. Shift currents are therefore only possible when inversion symmetry is broken and when there is a component of electric field along orthogonal crystal axes. For our experimental configuration, *L*_1*x*_(*t*) therefore requires an electric field component in the direction of the sample surface normal (i.e., along the *c*-axis, *ẑ* in Figure 1c), so it is not observed at normal incidence when photoexcited by either circularly or linearly polarized light. However, a significant contribution is observed for illumination with elliptically polarized light (see Section S9 in the Supporting Information), suggesting that LPGE can also be used to control the polarization of the emitted THz radiation. *C*_*x*_(*t*) is negligible even for elliptical polarization.

Following excitation with circularly polarized NIR photons, electrons are excited to higher energy states with different group velocities, creating an asymmetric momentum distribution in the helical Dirac cones that can lead to a photocurrent or “injection current” via CPGE. If this transfer of photon angular momentum is accompanied by a transfer of linear momentum, photocurrents can also be generated via the CPDE. The photogalvanic effect is forbidden in inversion-symmetric systems, as the net CPGE current vanishes on integration over *k*-space due to its rotational symmetry. In contrast, the photon drag effect can exist in any symmetric material but is related to the wavevector of light and vanishes at normal incidence.^73,74^ We therefore do not expect it to contribute to the emitted signal shown in Figure 2. For centrosymmetric WSMs, each Weyl node can produce both chirality-independent and chirality-dependent photocurrents. However, as the Weyl nodes have opposite tilt and chirality, the photocurrents will cancel.^31^ We therefore do not expect to observe any helicity-dependent currents for the centrosymmetric bulk single crystal at normal incidence. This is confirmed in Figure 2, where the *C*_*x*_(*t*) and *L*_1*x*_(*t*) terms were found to be negligible. To observe a non-zero CPGE in this system, the symmetry must be lowered by either applying an in-plane strain, magnetic field or by photoexcitation at oblique incidence.

CPGE is permitted for systems with a broken inversion symmetry, such as the non-centrosymmetric nanowire ensemble. By reducing the crystal symmetry from *C*_4*v*_ to *C*_4_ symmetry or breaking the TR symmetry, the DSM can be converted into a WSM with two pairs of Weyl nodes^59,67,75−77^ or driven as a Floquet–Weyl semimetal^43^ (see Section S3 in the Supporting Information). In this case, the time-reversal symmetry relates two Weyl nodes of the same chirality, and as the nodes have different tilts, there is a non-zero photocurrent^31^ for photoexcitation with circular polarization. However, despite fulfilling symmetry requirements, the net CPGE current can still vanish in these systems at normal incidence, as the Berry curvature vanishes for an in-plane spin distribution. Fitting of the experimental data for the non-centrosymmetric nanowire ensemble in Figure 3 shows both negligible THz emission and *C*_*x*_(*t*) contribution for circular polarization. This confirms that the nanowire ensemble has a pure Dirac linear dispersion, where the spins are planar: the photon spin is orthogonal to the 2D spin texture of the Dirac cone at normal incidence. A non-zero CPGE therefore requires the spins to be tilted out of plane, which can be achieved by varying the incident angle of the optical pump pulse.

### Polarization Dependence of THz Emission at Oblique Incidence

**Figure 4:**
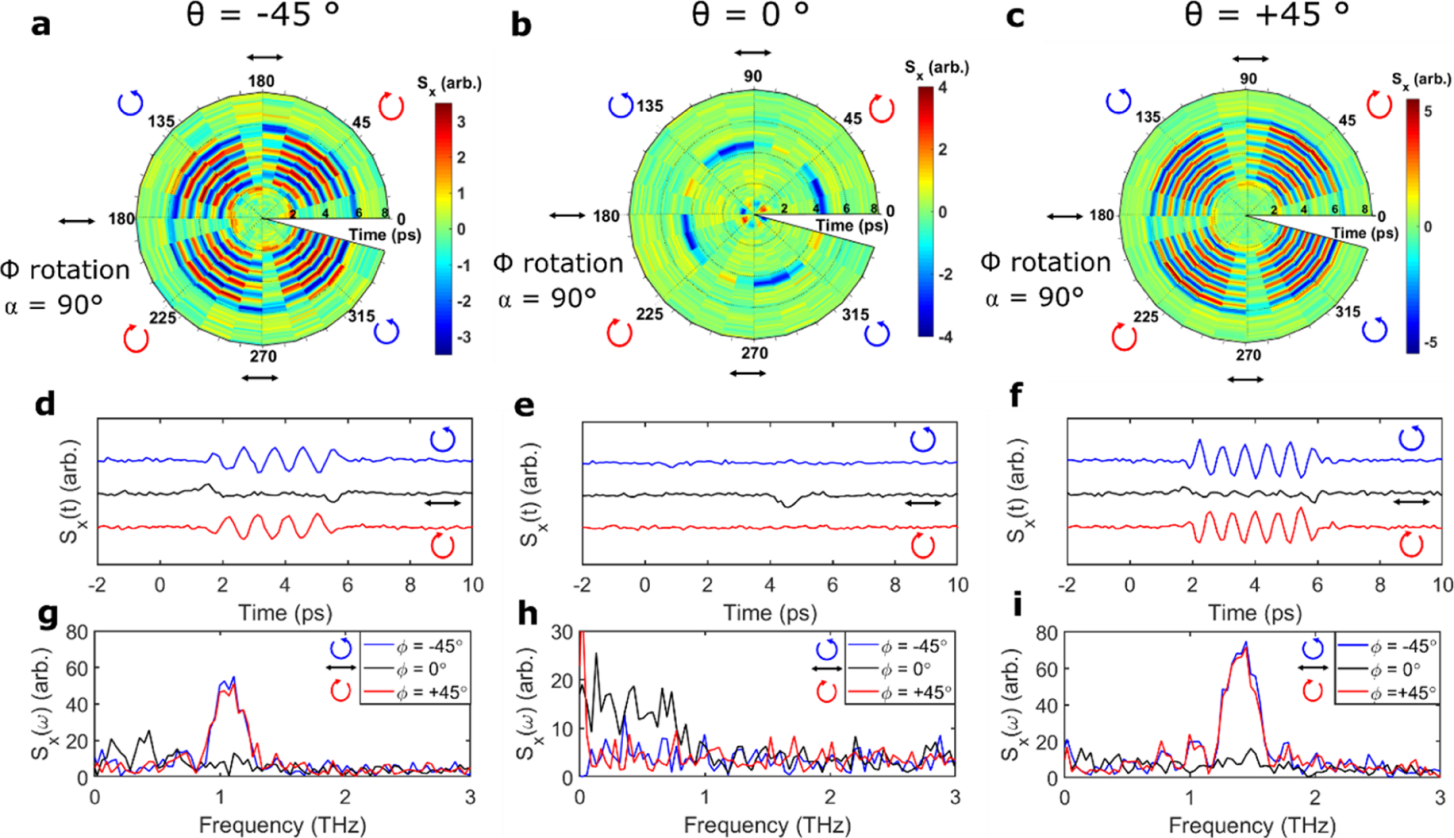
(a–c) Polar plots of THz waveforms as a function of polarization angle, φ, for the nanowire ensemble at incident angles of −45, 0, and +45° respectively. All data sets were taken at α = 90° to minimize the SOR contribution (Figure 1e). The time delay is plotted along the radius and the polarization angle against the circumference. The color bar represents the amplitude of the THz emission. (d–f) THz waveforms for the nanowire ensemble when excited with right-handed circularly (blue), linearly (black), and left-handed circularly polarized (red) NIR photons (i.e., for φ = 315, 0, +45° in (a–c) for θ = −45, 0, +45° respectively). (g–i) Corresponding spectra obtained by FFT of waveforms in (d).

We therefore next observe the polarization dependence of THz emission from the Cd_3_As_2_ nanowire ensemble when excited with NIR photons at oblique incidence. Figure 4a–c shows polar plots of the THz waveforms emitted at incident angles of θ = −45, 0, and +45° respectively. The emitted electro-optic signal was recorded at an azimuthal angle of α = 90° (see Figure 1f) with the nanowire axis aligned perpendicular to horizontal linear polarization (φ = 0°) to minimize linear absorption and contribution from bulk photocurrents. Figure 4d–f presents the time-domain trace of the emitted THz radiation when excited with NIR photons with right-handed circular (blue), horizontal-linear (black), and left-handed circular (red) polarization, with their corresponding FFT spectra shown in Figure 4g–i.

At normal incidence, there is negligible THz signal when the Cd_3_As_2_ nanowires are excited with circularly polarized photons, as seen previously, yet a small contribution remains when excited with linearly polarized photons owing to residual bulk PDE and thermoelectric currents in the nanowires. Similarly, a small THz emission signal was observed from the nanowires when photoexcited with linearly polarized photons at oblique incident angles of ±45°. However, when excited at these oblique angles with circularly polarized photons, a large electro-optic signal was observed, with an emitted THz amplitude 4 times higher than that observed for linear polarization. The emitted THz signal changes polarity with opposing NIR photon helicity, as expected for both CPGE and CPDE. It also switches polarity between positive and negative incident angles, which is a key feature of CPGE, CPDE, and PDE. Strikingly, multi-cycle THz pulses are emitted, leading to a narrowband source with a FWHM of ∼0.4 THz. For a positive incident angle (θ = +45°), the emission is centered at ∼1.40 THz, whereas a negative incident angle (θ = −45°) shifts the emission peak to a lower frequency of ∼1.07 THz. This asymmetry between negative and positive incident angles is attributed to the electron–hole asymmetry and asymmetry within the helical Dirac cone^77^ and provides an effective means for tuning the peak emission frequency via angle tuning of the nanowire ensemble.

**Figure 5:**
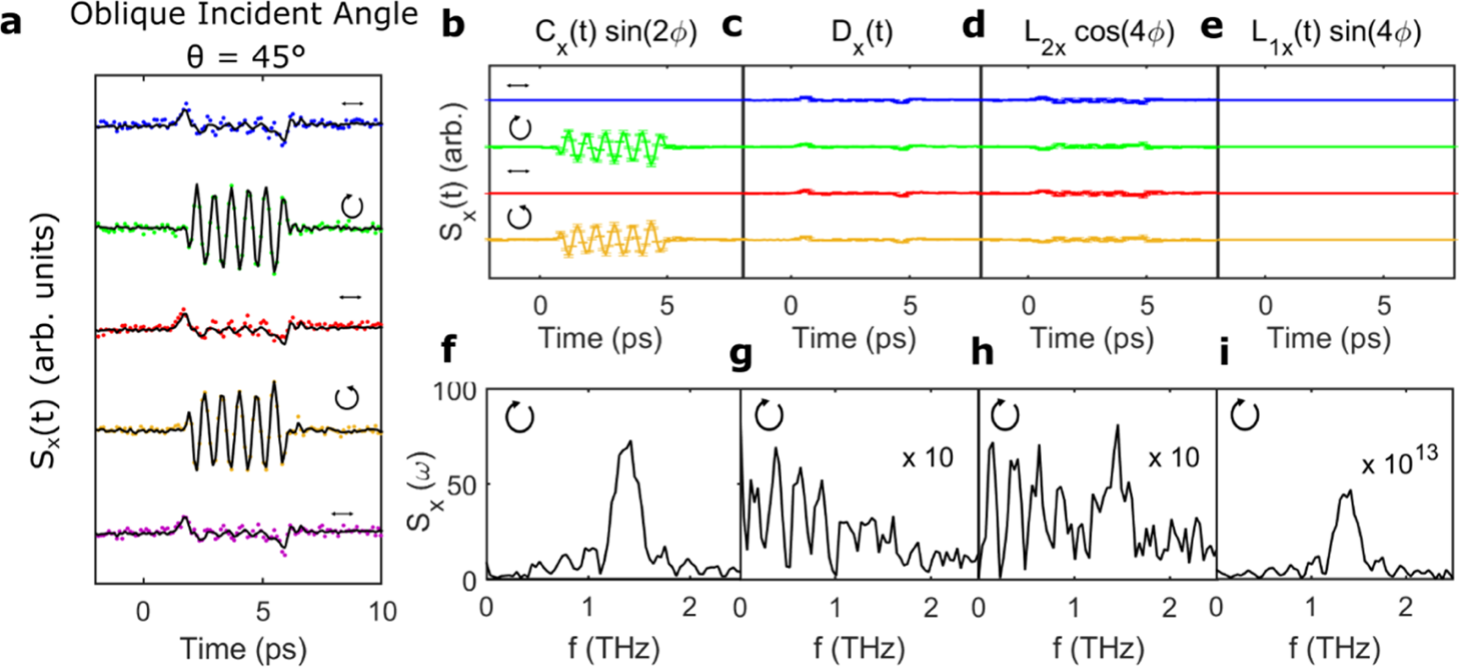
(a) Emitted THz waveforms for the nanowire ensemble at α = 90° when illuminated by photons with linear (φ = 0, 90, 180°) and circular (φ = 45, 135°) polarization at an oblique incident angle of 45°, respectively. The symbols indicate the experimental data, and the solid lines indicate the fitting result from eq 1. (b–e) Time-domain traces of contributions to the electro-optic signal, *S*_*x*_(*t*), from the terms *C*_*x*_(*t*) sin(2φ), *L*_1*x*_(*t*) sin(4φ), *L*_2*x*_(*t*) cos(4φ), and *D*(*t*) extracted from eq 1 for horizontal linearly (blue), right-handed circularly (green), vertical linearly (red), and left-handed circularly polarized (orange) optical pulses (i.e., φ = 0, +45, +90, +135°). The *y*-axes are all the same scale, and the extracted contributions are plotted with the 95% confidence intervals calculated from the fitting process. (f–i) Corresponding spectra obtained by the FFT of waveforms in (b–e) when the nanowire ensemble was illuminated with right-handed circularly polarized NIR photons.

To confirm the origin of the photocurrents responsible for this narrowband response, we again fit eq 1 and perform the same time-domain reconstruction as applied at normal incidence (Figure 3). Figure 5a shows the experimental (circles) and fitted (solid line) THz waveforms for example polarization angles. The extracted time-domain traces for all coefficients associated with each term in eq 1 are presented in Figure 5b–e, with their corresponding FFT spectrum displayed in Figure 5f–i. From inspection of the coefficients, the narrowband response, observed when the nanowires are photoexcited with circularly polarized NIR photons, appears to be primarily replicated by *C*_*x*_(*t*) and a result of helicity-dependent photocurrents. We note that for photoexcitation at large oblique incident angles, these helicity-dependent currents could be due to both the CPGE and the CPDE. For our experimental configuration, it is challenging to distinguish between the two effects without assessing the wavelength dependence of the emission,^78^ which is beyond the scope of this work. However, we expect CPGE to dominate our emitted signal, as our optical photoexcitation primarily promotes interband transitions.^74^*L*_1*x*_(*t*) also replicates the shape of the waveform, yet its extracted amplitude is 13 orders of magnitude smaller than the *C*_*x*_(*t*) contribution and is therefore negligible. However, when the nanowires are excited with elliptically polarized photons (φ = 15, 30, 60, 75°), the magnitude of the *L*_1*x*_(*t*) contribution becomes comparable to that of *C*_*x*_(*t*) (see Figure S10 in the Supporting Information), so helicity-independent photocurrents via LPGE cannot be ruled out. For spin–orbit coupled quantum well systems, CPGE and LPGE photocurrents have been theoretically predicted to have equal magnitude and be related to the Berry phase, with CPGE proportional to the Berry curvature and LPGE linked to the Berry connection. While this points to a topological origin, it is worth noting that the 1.55 eV photon energy of our optical pulses is too high to probe direct transitions within the Dirac cone. In contrast, we probe optical transitions from/to the Dirac band to/from the bulk bands and will therefore observe photocurrents arising from both the bulk and Dirac cones. Similarly, asymmetric scattering off phonons and defects can also contribute to the LPGE. Although we have set the azimuthal angle and utilized low photoexcitation fluences to reduce sample heating, bulk thermal effects and linear absorption, bulk currents, *D*(*t*) and *L*_2*x*_(*t*), still contribute to the signal, and we cannot rule out that our emission contains a small D_x_ contribution from thermoelectric currents due to the Seebeck effect.^79^ However, they display a broadband response that is 10 times smaller than the contribution from *C*_*x*_(*t*). For oblique incidence, helicity-dependent currents therefore dominate and can be used to provide a narrowband THz source.

**Figure 6:**
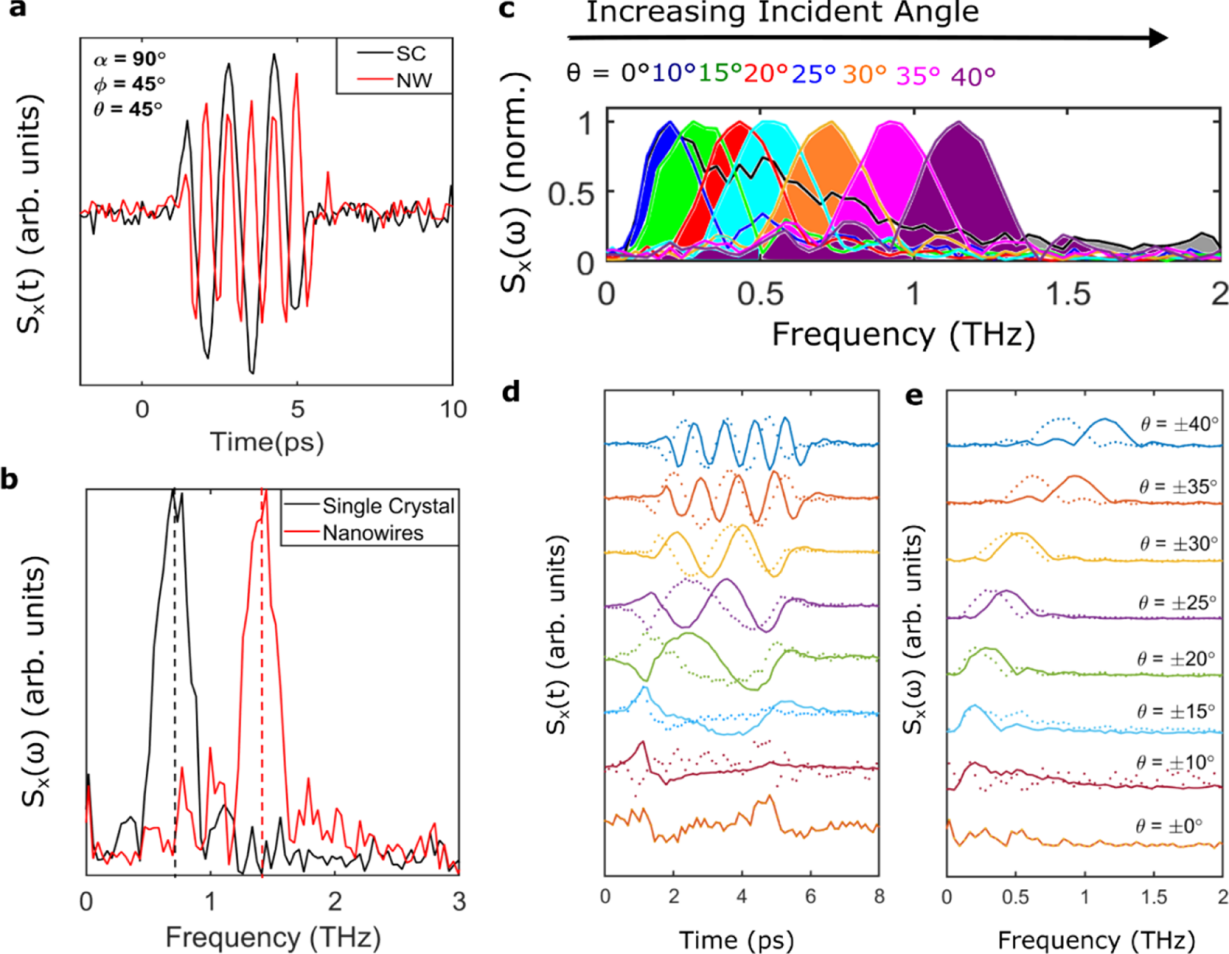
(a) Emitted THz waveforms for the bulk crystal (black) and nanowire ensemble (red) at α = 90° when illuminated with right-handed circularly polarized NIR photons (φ = 45°) at an oblique incident angle of 45°. (b) Corresponding spectra obtained by FFT of waveforms in (a). (c) Normalized FFT amplitude of the emitted THz waveform for the nanowire ensemble at varying incident angles from 10 to 40°. The narrowband THz emission is blue-shifted as the incident angle is increased. (d) THz waveforms and (e) corresponding spectra obtained via FFT for the nanowire ensemble at varying incident angles. Solid lines represent positive angles, and dotted lines indicate negative angles.

One exciting aspect of the observed THz emission from the non-centrosymmetric Cd_3_As_2_ nanowires when excited by a circularly polarized laser pulse at oblique incidence is the capability to tune the emission frequency by changing the angle of the incident light. Figure 6c shows the normalized FFT amplitude of the emitted THz signal for positive incident angles ranging from θ = 10 to 40°. For all angles above 10°, narrowband emission is observed, and the generated THz spectrum exhibits FWHM within the range of 0.33–0.48 THz. The peak emission frequency increases from 0.21 to 1.40 THz as the incident angle from θ = 15 to 40°. The time-domain waveforms and corresponding FFT spectra for both negative (dotted lines) and positive incident angles (solid lines) are shown in Figure 6d,e.

As previously observed, there is a clear asymmetry between negative and positive angles, with negative angles displaying a lower-peak emission frequency. For the emission from transient currents, the peak frequency of terahertz pulses is inversely proportional to the charge-carrier relaxation time. As the incident angle is varied (θ → −θ), the momentum of light surveys a different part of the band structure (*k*_*x*_ → −*k*_*x*_). The Dirac cones within Cd_3_As_2_ are asymmetric about the nodal points and also have an electron–hole asymmetry.^77^ We therefore attribute the change in the peak emission frequency to differing charge carrier relaxation times within the Dirac cone and between electrons and holes. A similar change in the peak emission frequency is also observed when comparing the centrosymmetric single crystal and non-centrosymmetric nanowire ensemble response. The THz waveform (Figure 6a) and corresponding FFT spectrum (Figure 6b) emitted for the single crystal (black) and nanowire ensemble (red) when excited with circularly polarized light (φ = 45°) at θ = 45°, α = 90° have a comparable amplitude. However, the centrosymmetric single crystal has a lower peak emission frequency, indicating a longer carrier relaxation time compared to the non-centrosymmetric nanowire ensemble. Previous studies have shown that single crystals display metallic behavior, whereas the Cd_3_As_2_ nanowire ensemble displays semiconducting behavior.^80^ The size quantization caused by the nanowire geometry can also provide size-quantized sub-bands within the conduction that exhibit smaller relaxation times. This provides another means for controlling THz emission, as the relaxation time and thereby the peak emission frequency can be tailored by altering the nanowire geometry.

## Conclusion

In conclusion, our work demonstrates helicity-dependent THz emission from the topological DSM Cd_3_As_2_. By changing the polarization of the optical drive pulse from linear polarization to circular polarization, both broadband and narrowband THz emission can be achieved. Under illumination by NIR photons with linear polarization at normal and oblique incidence, the photocurrent response was dominated by bulk thermal photocurrents, exhibiting broadband helicity-independent THz emission with spectral extension out to ∼1 THz. However, when the system is driven with circularly polarized NIR pulses at an oblique incidence, a narrowband response was observed. This THz emission changed polarity with opposing photon helicity and incident angle, identifying helicity-dependent currents as its origin. The peak emission frequency could also be tuned by varying the incident angle, ranging from 0.21 to 1.40 THz over an angle range of 15–40° for the nanowire ensemble. The peak emission frequency was also found to be different for the centrosymmetric single crystal and non-centrosymmetric nanowire ensemble, hinting that the size quantization of the nanowire geometry could allow further control of the peak emission frequency. We therefore highlight Cd_3_As_2_ nanowires as a promising, novel THz source that can be optically pumped by Ti:sapphire laser pulses (1.55 eV) and optically switched between broadband and narrowband operations with all-optical polarization control.

## Methods

### Terahertz Emission Spectroscopy

The terahertz emission experiments presented in this article were carried out using optical NIR pulses to photoexcite the sample and electro-optic sampling for the detection of the emitted THz radiation. The optical NIR pulses with 35 fs pulse duration and a central wavelength of 800 nm were generated at a repetition rate of 5 kHz by an amplified Ti/sapphire laser with an average power of 4 W. Each pulse was separated into two beam paths: one acting as the optical pump to photoexcite the sample and one as the gate beam (∼1.6 μJ/pulse) for electro-optic detection. To obtain a range of photoexcitation fluences, the optical pump beam was attenuated by neutral density filters. A half-wave plate was inserted into the optical pump beam before it illuminated the sample to vary the pump laser polarization state between photoexcitation with horizontal (φ = 0°) and vertical (φ = 90°) linearly polarized photons. To photoexcite the sample with circularly and elliptically polarized NIR photons, a quarter-wave plate was also inserted into the optical pump beam path after the half-wave plate. Following photoexcitation, the emitted THz pulse was collected in a transmission geometry and focused onto the electro-optic detection crystal with the gate beam by two parabolic mirrors. This optical path (from the sample to the detector) was placed under vacuum to avoid absorption of the emitted THz radiation by atmospheric water vapor. Electro-optic sampling of the emitted THz pulse was conducted via a 1 mm-thick (110) ZnTe crystal, where the THz electric field, *E*, was measured by recording the voltage signal from a balanced photodiode circuit through a lock-in amplifier referenced to a chopper at 2.5 kHz (half the laser repetition rate) placed in the optical pump beam path. The emitted THz electric field waveform was therefore traced by varying the time delay between the optical pump beam and the gate beam and by measuring the voltage signal from the balanced photodiodes. The emitted THz spectrum was obtained by taking the absolute Fourier transform of the measured THz waveform. All measurements were conducted at room temperature.

### Sample Fabrication and Characterization

The bulk single Cd_3_As_2_ crystal was grown inside a two-zone furnace from stoichiometric amounts of Cd and As elements. A detailed description of the growth process can be found in the Supporting Information and in ref (62). The Cd_3_As_2_ nanowires were grown via chemical vapor transport in a horizontal tube furnace. Each batch of nanowire samples was characterized by scanning electron microscopy and energy dispersive spectroscopy. The crystal structure was determined by X-ray diffraction and transmission electron microscopy. The self-catalyzed growth process and structural characterization are described in more detail in the Supporting Information and in ref (61).

## Supporting Information Available

Additional details of the sample growth; THz emission experimental setup and data modeling; summary of THz emission mechanisms and topological band structures; detailed plots of THz emission waveforms for both samples at all azimuthal, polarization, and incident angles; and comparison of narrowband emission from the Cd_3_As_2_ nanowire ensemble to the standard ZnTe THz emitter (PDF)

## Notes

The data sets supporting the findings reported in this paper are openly available from the University of Manchester FigShare repository.
