# Supplementary material for: Narrowband, Angle-Tunable, Helicity-Dependent Terahertz Emission from Nanowires of the Topological Dirac Semimetal Cd3As2
Source: ACS Photonics. 2023 Apr 3;10(5):1473–84. doi: 10.1021/acsphotonics.3c00068 (PMC10197169; doi:10.1021/acsphotonics.3c00068)
Supplement: Supplementary file 1 — ph3c00068_si_001.pdf [file ph3c00068_si_001.pdf]

Supporting Information for 'Narrowband, angle-tuneable, helicity-dependent terahertz emission from nanowires of the topological Dirac semimetal Cd_3As_2'

Jessica L. Boland[1], Djamshid A. Damry[1], Chelsea Q. Xia[2], Piet Schoenherr[2], Dharmalingam Prabhakaran[2], Laura M. Herz[2], Thorsten Hesjedal[2], Michael B. Johnston[2*]*

[1]Photon Science Institute, Department of Electrical and Electronic Engineering, University of Manchester, Manchester M13 9PL, UK

[2]Department of Physics, University of Oxford, Clarendon Laboratory, Parks Road, Oxford OX1 3PU, UK

17 pages, 13 figures, 2 tables

1. Growth mechanisms and crystal structure for single crystals

High-quality Cd_3As_2 single crystals were synthesized by placing stoichiometric amounts of high-purity (>99.99%) Cd and As elements inside an evacuated carbon-coated quartz tube, which was sealed in another evacuated tube for extra protection. The two tubes were then placed inside a two-zone furnace and heated very slowly at a rate of 25°C/h to 850°C to avoid any cracking. After 24 h, the furnace was cooled down to 550°C at a rate of 2°C/h and finally cooled down to room temperature at a rate of 60°C/h. Single crystals of size up to 5 mm were separated and characterised for further experiments[1]. The bulk single crystal used in this study was approximately 1mm^3 in size. The bulk single crystal has a tetragonal crystal structure belonging to the centrosymmetric $I4_1/acd$ space group, as shown in Ref. 1.

2. Growth mechanism and crystal structure of the nanowire ensemble

The Cd_3As_2 nanowires were grown in a self-catalysed process from a Cd_3As_2 precursor in a horizontal tube furnace using N_2 as the carrier gas. The growth window is relatively narrow, and the most characteristic growth feature is the exclusive growth from Cd_3As_2 clusters. In contrast to most III-V nanowire materials, the metal Cd has a higher vapour pressure than the chalcogen As, which leads to a different growth mode as one cannot assume the usual As-rich conditions. For illustrating the vapour-solid growth of Cd_3As_2, it is instructive to think about it as the inverse of its vaporisation. When Cd_3As_2 is heated, Cd evaporates first and leaves an As rich surface behind. The Cd vacancies can be replenished by Cd diffusing from the bulk if the surface-to-volume ratio is small. Inversely, if a given crystal is exposed to Cd and As vapour, it will grow an As-rich layer that gradually incorporates incoming Cd atoms. Therefore, the tip of nanowires and the surface layer of clusters are As-rich. Further, growth also proceeds by crystallization on the side walls, judging from the tapering of the nanowires which are narrow at the top and wider at the root. Details of the growth mechanisms are summarised in detail in Ref. 2. The growth process resulted in a nanowire distribution with an average diameter of 100 nm and an average length of 15 µm.

The structural properties of the Cd_3As_2 nanowires were determined by powder x-ray diffraction (XRD) and transmission electron microscopy (TEM) (for details see Ref. 2). α-Cd_3As_2 crystalizes in the non-centrosymmetric space group $I4_1cd$ (low temperature phase), as confirmed by fits to our powder XRD data using TOPAS (Bruker AXS. Topas V 4.2 (2009)).. The tetragonal unit cell measures a = 12.67 Å and c = 25.48 Å and can be roughly visualised as consisting of cubic close-packed As ions and Cd-As_4 tetrahedra[3]. The [112] interplanar spacing determined in TEM of 0.73 nm agrees well with the calculated value of 0.732 nm.

Following growth, the nanowires were transferred onto z-cut quartz substrates by preferentially rubbing them in one direction. This resulted in a dense nanowire matrix with the

nanowire axis predominantly aligned in one direction. Figure S1 shows an optical microscope image of the nanowire sample.

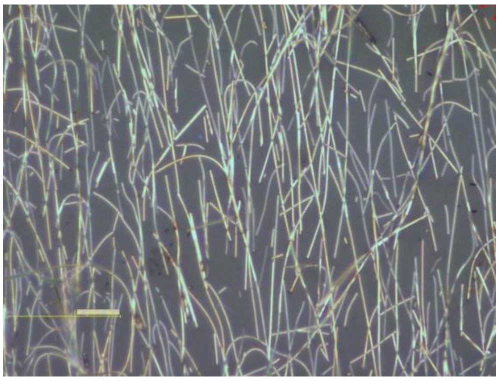

Figure S1. Optical microscope of Cd_3As_2 nanowire ensemble. Scale bar is 20 µm.

3. Bandstructure for DSMs, Type-I WSMs and Type-II WSMs

Figure S2 shows a schematic diagram of a typical bandstructure for topological semimetals.

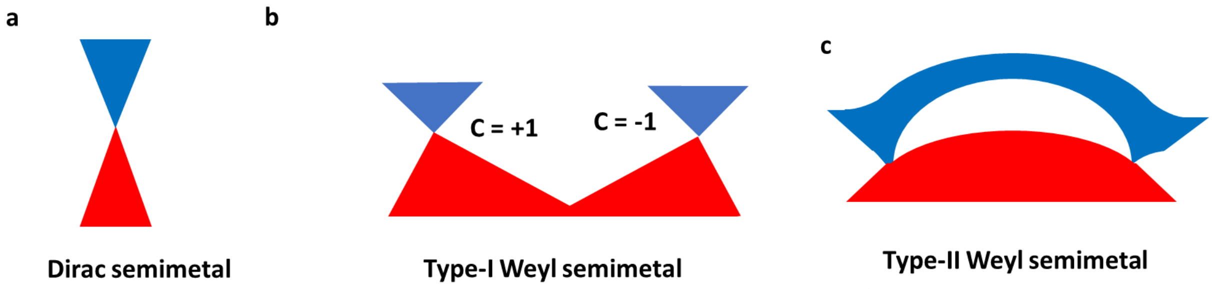

Figure S2. Schematic diagram of a typical bandstructure for **a,** a Dirac semimetal; **b,** a type-I Weyl semimetal and **c,** a type-II Weyl semimetal. The valence band is represented in red and the conduction band in blue. The type-I Weyl semimetal has opposite chiral charges at two degenerate Weyl points, which is indicated by C = +1 and C = -1 on the diagram.

4. THz emission spectroscopy system

Figure S3 shows a schematic diagram of the THz emission spectroscopy system, which is described in the methods section of the main text.

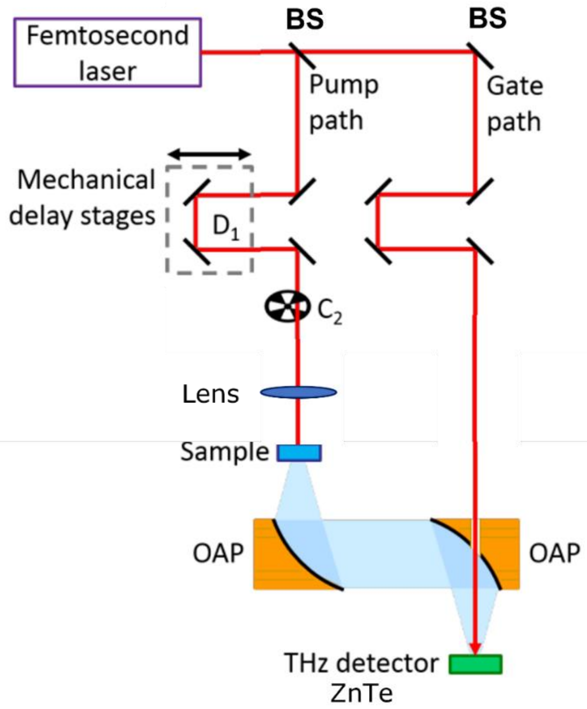

Figure S3. Schematic diagram of the experimental setup. A femtosecond pulse is split into two beam paths: a gate beam path and a pump beam path. The gate beam travels straight to the THz detector (1 mm ZnTe crystal). The pump beam is directed to the sample via a delay stage, where it induces THz emission. The THz emission is collected by a parabolic mirror, where it is collimated and then focused onto the THz detector for electro-optic sampling with the gate beam.

5. Physical mechanisms for THz generation

Table S1 shows the physical mechanisms behind different THz generation processes, alongside conditions on crystal structure and photoexcitation[4,5]. For the experiments presented in the manuscript, two samples were examined: a bulk centrosymmetric crystal and a non-centrosymmetric nanowire ensemble. Both were photoexcited above the bandgap with ~1.5 eV optical pump pulses. At normal incidence with linear polarisation, some of the listed mechanisms in Table S1 can be immediately discounted. For THz generation from the photo-Dember and surface-field effects, the current generated would be normal to the surface and therefore not radiate into free-space at normal incidence[6,7].

Table S1: Description of physical mechanisms behind THz emission mechanisms

	Bulk Optical Rectification	Surface Optical Rectification	Surface Field	Photo-Dember Effect
	$\vec{E}_{\text{THz}} \sim \frac{\partial^2 \vec{P}}{\partial t^2}$<br><br>$P^{(2)}_{jk} = \sum_{jk} \varepsilon_0 \chi^{(2)eff}_{ijk}(\Omega, \omega + \Omega, -\omega) E_j(\omega + \Omega) E^*_k(\omega)$	$\vec{E}_{\text{THz}} \sim \frac{\partial^2 \vec{P}}{\partial t^2}$<br><br>$P^{(2)}_{jk} = \sum_{jk} 3\varepsilon_0 \chi^{(3)eff}_{ijkl}(\Omega, \omega + \Omega, -\omega) E^{surf}_z E_j(\omega + \Omega) E^*_k(\omega)$	$\vec{E}_{\text{THz}} \sim \frac{\partial \vec{J}}{\partial t}$	$\vec{E}_{\text{THz}} \sim \frac{\partial \vec{J}}{\partial t}$
Mechanism	Nonlinear polarisation induces THz field by difference-frequency generation	Surface depletion field can break symmetry to induce nonlinear polarisation and generate THz field by difference-frequency generation	Band-bending at surface induces a depletion field, which leads to a photocurrent surge that emits THz radiation	Difference in electron and hole mobility leads to separation of photoexcited electrons and holes at the surface, creating a Dember field that leads to diffusion and drift photocurrent
Type of process	2nd order nonlinear process	3rd order nonlinear process	Linear process	Linear process
What is it probing?	Nonlinear polarisation of 'virtual' carriers	Nonlinear polarisation of 'virtual' carriers and surface depletion field	Transient photocurrent of real carriers, band-bending, nature of surface field, carrier transport dynamics	Transient photocurrent of real carriers, Dember field, change in mobilities
Photoexcitation	Below bandgap excitation	Below bandgap excitation	Above bandgap excitation	Above bandgap excitation
Crystal Structure	Non-centrosymmetric	Centrosymmetric	Wide-bandgap materials with band-bending	Narrow-bandgap materials
Ways to test	Rotation of azimuthal angle, as should follow crystal symmetry (should also match SHG)	Rotation of azimuthal angle, as should have 3-fold symmetry	Polarity of THz radiation changes with doping. Surface modification (e.g., passivation)	Amplitude should increase at oblique angles.

	Linear Photogalvanic Effect	Circular Photogalvanic Effect	Photon Drag Effect	Magnetism
	$\vec{E}_{\text{THz}} \sim \frac{\partial \vec{J}}{\partial t}$<br>$J_i = \chi_{ijk} E_j E_k^*$<br>$\chi_{ijk}$ and T_{ijkl} are 3rd rank and 4th rank tensor respectively	$\vec{E}_{\text{THz}} \sim \frac{\partial \vec{J}}{\partial t}$<br>$J_i = \sum_j \gamma_{ij}\, i(\boldsymbol{E} \times \boldsymbol{E})_j$<br>$\gamma_{ij}$ is a 3rd order pseudo tensor for CPGE	$\vec{E}_{\text{THz}} \sim \frac{\partial \vec{J}}{\partial t}$<br>$J_i = T_{ijkl}\{E_j(\nabla_l E_k^*) - (\nabla_l E_j)E_k^*\} = T_{ijkl} q E_j E_k^*$	$\vec{E}_{\text{THz}} \sim \frac{\partial^2 \vec{M}}{\partial t^2}$
Mechanism	Spatial charge transfer during the transition from valence band to conduction band under photoexcitation from linear polarised light	Asymmetric distribution of carrier in *k*-space due to excitation with circularly-polarised light	Momentum transfer from incident photons to electrons near the surface in the penetration depth	Circularly polarised light injects spin-polarised electrons and holes and induces magnetisation due to spin imbalance
Type of process	2nd order nonlinear process	2nd order nonlinear process	Linear with increasing fluence	Linear process
What is it probing?	Transient photocurrent of real carriers along polar direction	Transient photocurrent of real carriers	Transient photocurrent of real carriers in direction of incident light	Transient photocurrent of real carriers
Photoexcitation	Above bandgap excitation	Above bandgap excitation	Above bandgap excitation	Above bandgap excitation
Crystal Structure	Non-centrosymmetric, any materials with a polar axis or without inversion symmetry	Non-centrosymmetric (only crystals without inversion symmetry or at surface)	Usually in doped semiconductors and metal (i.e., high free carrier concentration)	
Ways to test	Photocurrent depends on crystalline symmetry<br>HWP rotation, 2nd order dependence on electric field, no difference for LH or RH	Excited by circularly polarised light and polarity change for LH and RH and different incident angles (± 45°), quadratic with E field	Polarity change for linear polarised light at different incident angles (± 45°)	Emitted THz radiation proportional to laser intensity, polarity changes for LH and RH

As the bulk crystal is centrosymmetric, any optical rectification response observed must also be due to the surface, whereas the nanowire ensemble can portray a rectification response from the bulk. However, other mechanisms are expected to dominate due to above bandgap photoexcitation. For linear polarisation, shift currents due to LPGE are expected for the nanowire ensemble, as Cd_3As_2 is a polar material without inversion symmetry. For both the single crystal and nanowire ensemble, we also expect a contribution from the photon drag effect, as Cd_3As_2 has a high carrier concentration. For circular polarisation, injection currents via CPGE are only predicted at normal incidence for the nanowire ensemble, due to its lack of inversion symmetry.

6. Dependence of the THz emission mechanisms on crystal orientation, incident angle and polarisation

Table S2 shows how the emission amplitude depends on crystal orientation (azimuthal angle, α), helicity (QWP angle, ϕ), linear polarisation angle (HWP angle, ϕ), and incident angle (ϴ).

Table S2: Polarisation, incident and azimuthal angle dependence of THz emission mechanisms

	**α**<br>**Crystal orientation**	**ϕ**<br>**Polarisation angle (QWP angle)**	**ϕ**<br>**Polarisation angle (HWP angle)**	**ϴ**<br>**Incident angle**
**CPGE**	Independent	$sin\,2\phi$	Independent	Polarity change
**LPGE**	Independent	$sin\,4\phi$	$\cos 2\phi$	Polarity change
**PDE**	Dependent	$\cos 4\phi$	$\cos 2\phi$	Polarity change
**OR**	Dependent	$cos\,4\phi$	$\cos 2\phi$	No polarity change

7. Azimuthal angle dependence of the THz emission at normal incidence

Figure S4 and Figure S5 show the time-domain traces and corresponding FFT spectrum of the emitted THz pulses for both the single crystal and nanowire ensemble when photoexcited at different azimuthal angles with NIR photons with linear polarisation at normal incidence. The

horizontal component of the emitted THz pulses is detected and is minimised at α = 90° for both samples.

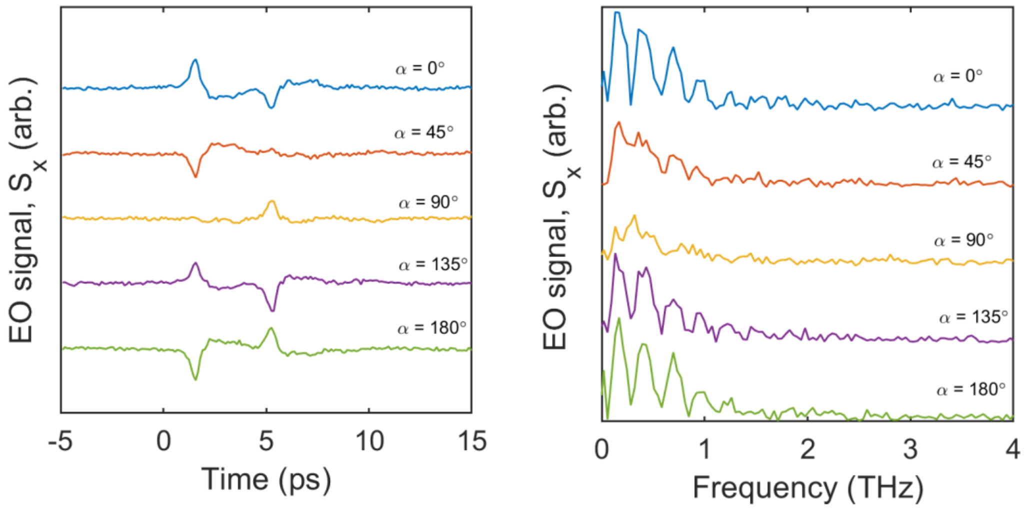

Figure S4. Time-domain traces of emitted THz pulses (left) and their corresponding FFT spectra (right) for the single crystal when photoexcited at normal incidence with NIR photons with linear polarisation at varying azimuthal angles. The traces are offset for clarity but are plotted on the same y-axis scale.

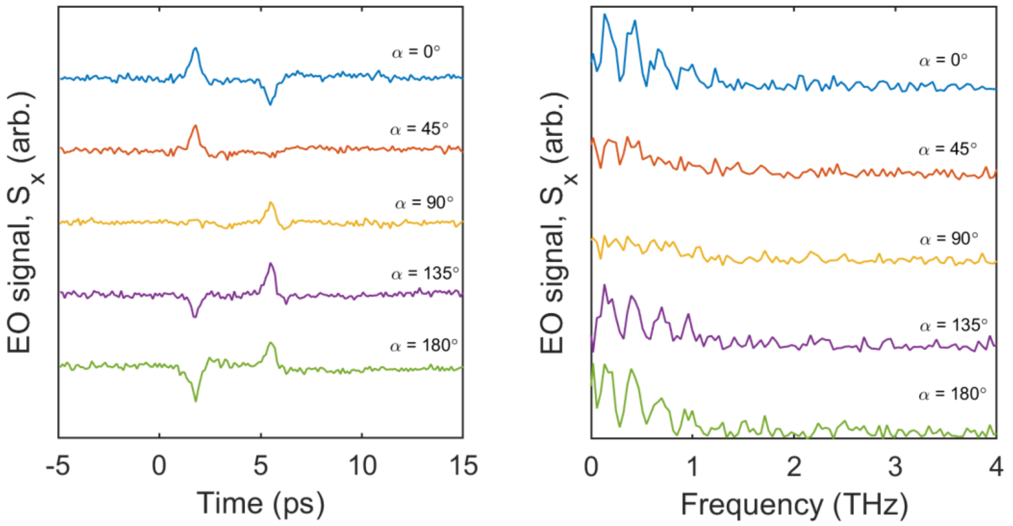

Figure S5. Time-domain traces of the emitted THz pulses (left) and their corresponding FFT spectra (right) for the nanowire ensemble when photoexcited at normal incidence with NIR photons with linear polarisation at varying azimuthal angles. The traces are offset for clarity but are plotted on the same y-axis scale.

8. Time-domain reconstruction of the THz emission mechanisms for bulk crystal at normal incidence

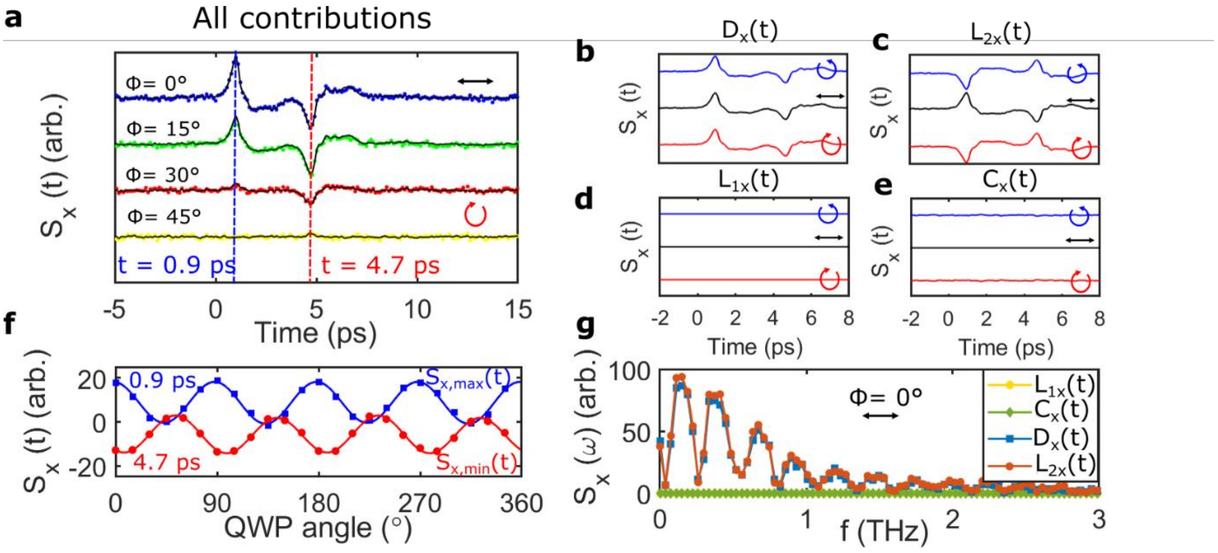

Figure S6 a, Emitted THz waveforms for the single crystal under linearly- (ϕ = 0°), elliptically- (ϕ = 15°, 30°), and circularly-polarised (ϕ = 45°) illumination at normal incidence for an azimuthal angle α = 180° (maximum THz emission). The symbols indicate the experimental data and the solid lines the fitting result from Equation (1). **b-e,** Time-domain traces of coefficients, C′(t), L′(t), D′(t) and O(t) extracted from Equation (1) for right-handed circularly- (blue), linearly- (black) and left-handed circularly-polarised (red) optical pulses (i.e., ϕ = -45°, 0°, +45°). These coefficients represent CPGE, LPGE, PDE and OR contributions to THz emission, respectively. **f,** THz amplitude as function of polarisation angle, ϕ at a time delay corresponding to dashed lines in **a,** t = 0.9 ps (blue) and 4.7 ps (red). **g,** Corresponding spectra for CPGE (green diamonds), LPGE (yellow stars), PDE (red circles) and OR (blue squares) obtained by FFT of extracted coefficient amplitudes in b-e for illumination under linearly-polarised light at normal incidence.

Figure S6a shows the time-domain emitted THz waveforms for the bulk single crystal at normal incidence taken at an azimuthal angle of 180°. At this crystal orientation, the observed THz emission was at a maximum. The extracted time domain traces from fitting Equation (1) in the main manuscript to the experimental data (solid lines in Figure S6a) are presented in Figure S6b-e. Contributions from CPGE and LPGE ($C_x(t)$ and $L_{1x}(t)$) are negligible, as

expected for a centrosymmetric crystal. The overall emitted response is replicated by contributions from bulk photothermal currents, $D_x(t)$ and $L_{2x}(t)$. $L_{2x}(t)$ represents the photocurrent due to the photon drag effect and switches polarity with for excitation with linear and circular polarisation.

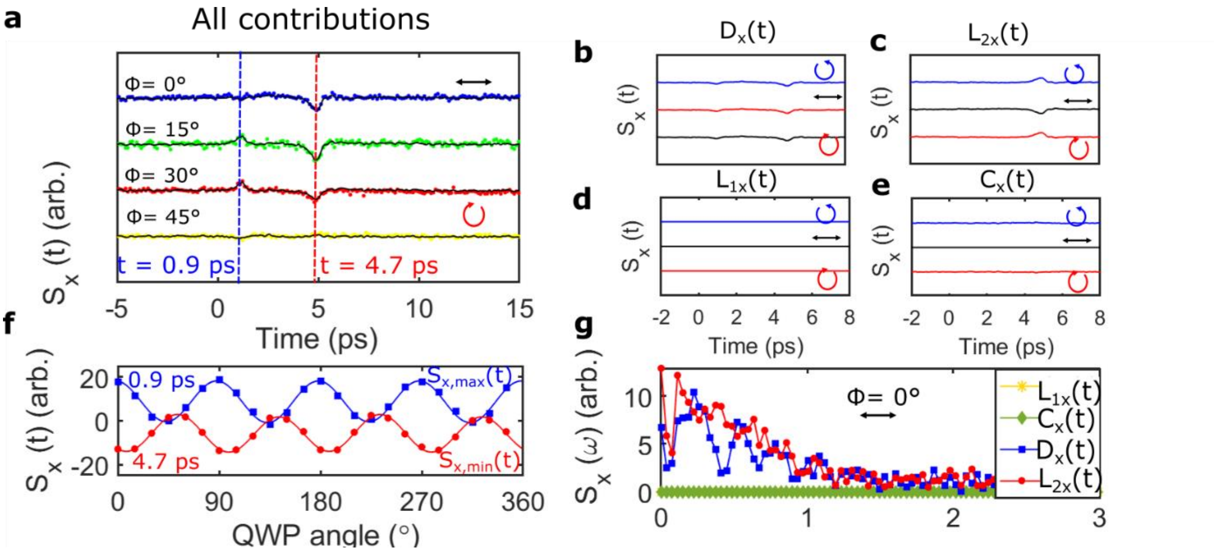

Figure S7 a, Emitted THz waveforms for the single crystal under linearly- (ϕ = 0°), elliptically- (ϕ = 15°, 30°), and circularly-polarised (ϕ = 45°) illumination at normal incidence for azimuthal angle, α = 90° (minimum THz emission). The symbols indicate the experimental data and the solid lines the fitting result from Equation (1). **b-e,** Time-domain traces of coefficients, C′(t), L′(t), D′(t) and O(t) extracted from Equation (1) for right-handed circularly- (blue), linearly- (black) and left-handed circularly-polarised (red) optical pulses (i.e., ϕ = -45°, 0°, +45°). These coefficients represent CPGE, LPGE, PDE and OR contributions to THz emission, respectively. **f,** THz amplitude as function of polarisation angle, ϕ at a time delay corresponding to dashed lines in **a,** t = 0.9 ps (blue) and 4.7 ps (red). **g,** Corresponding spectra for CPGE (green diamonds), LPGE (yellow stars), PDE (red circles) and OR (blue squares) obtained by FFT of extracted coefficient amplitudes in b-e for illumination under linearly-polarised light at normal incidence.

Figure S7 shows the same experimental data for bulk single crystal at normal incidence but now taken at an azimuthal angle of 90°, where the observed THz emission was at a minimum. For this crystal orientation, the contribution from polarisation-independent currents and

rectification is minimised. Again, the THz response is dominated by bulk photothermal currents and is replicated by $D_x(t)$ and $L_{2x}(t)$. We also notice that the emitted signal from photocurrent due to the photon drag effect, $L_{2x}(t)$, is delayed in time compared to the signal from other bulk currents and rectification, $D_x(t)$. This again hints at the two contributions being due to different photocurrents with different relaxation times. We suggest that these differing relaxation times could be due to the electron-hole asymmetry in Cd_3As_2, or from relaxation between bands and to/from a band into/from the Dirac cone.

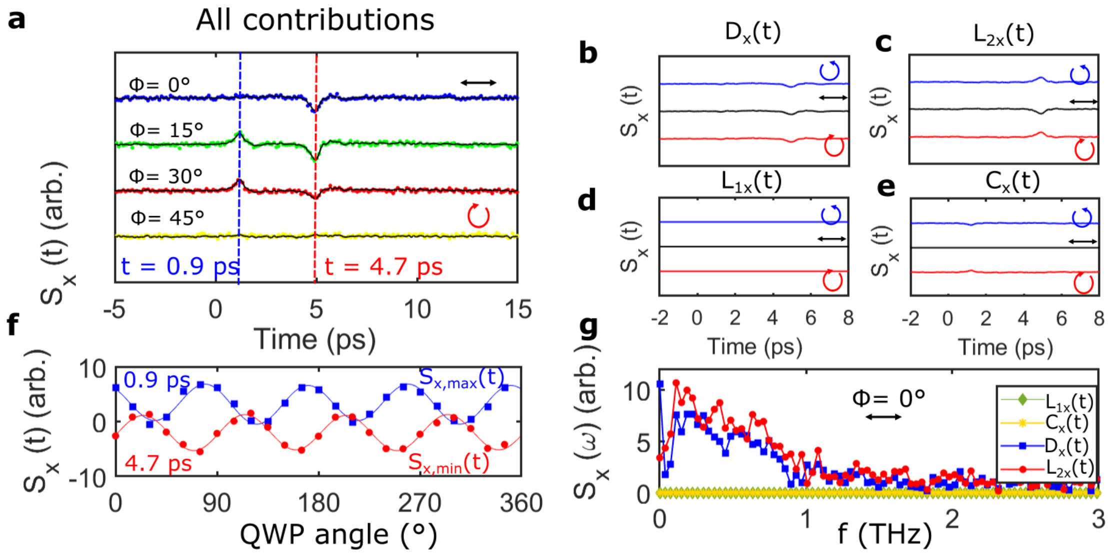

Figure S8. **a,** Emitted THz waveforms for the nanowire ensemble under linearly- (ϕ = 0°), elliptically- (ϕ = 15°, 30°), and circularly-polarised (ϕ = 45°) illumination at normal incidence for azimuthal angle, α = 90° (minimum THz emission). The symbols indicate the experimental data and the solid lines the fitting result from Equation (1). **b-e,** Time-domain traces of coefficients, C′(t), L′(t), D′(t) and O(t) extracted from Equation (1) for right-handed circularly- (blue), linearly- (black) and left-handed circularly-polarised (red) optical pulses (i.e., ϕ = -45°, 0°, +45°). These coefficients represent CPGE, LPGE, PDE and OR contributions to THz emission, respectively. **f,** THz amplitude as function of polarisation angle, ϕ at a time delay corresponding to dashed lines in **a,** t = 0.9 ps (blue) and 4.7 ps (red). **g,** Corresponding spectra for CPGE (green diamonds), LPGE (yellow stars), PDE (red circles) and OR (blue squares) obtained by FFT of extracted coefficient amplitudes in b-e for illumination under linearly-polarised light at normal incidence.

Figure S8a shows the time-domain emitted THz waveforms for the nanowire at normal incidence taken at an azimuthal angle of 90°. At this crystal orientation, the observed THz emission was at a minimum and the effects from linear absorption reduced. As observed in the main manuscript, contributions from CPGE and LPGE ($C_x(t)$ and $L_{1x}(t)$) are again negligible. This is unexpected, as the nanowire ensemble is non-centrosymmetric and CPGE is allowed. However, for an in-plane spin distribution, the photocurrents will cancel. For pure Dirac linear dispersion, the Berry curvature will also vanish, leading to zero photocurrent. We therefore conclude that our system does exhibit a pure Dirac linear dispersion and in-plane spin distribution. The overall emitted response is replicated by contributions from bulk photothermal currents, $D_x(t)$ and $L_{2x}(t)$. A similar delay in time for $L_{2x}(t)$ compared to $D_x(t)$ is also observed, again suggesting the presence of mechanisms with different relaxation times.

9. Time-domain reconstruction of $L_{1x}(t)$ contribution at normal incidence with elliptical polarisation

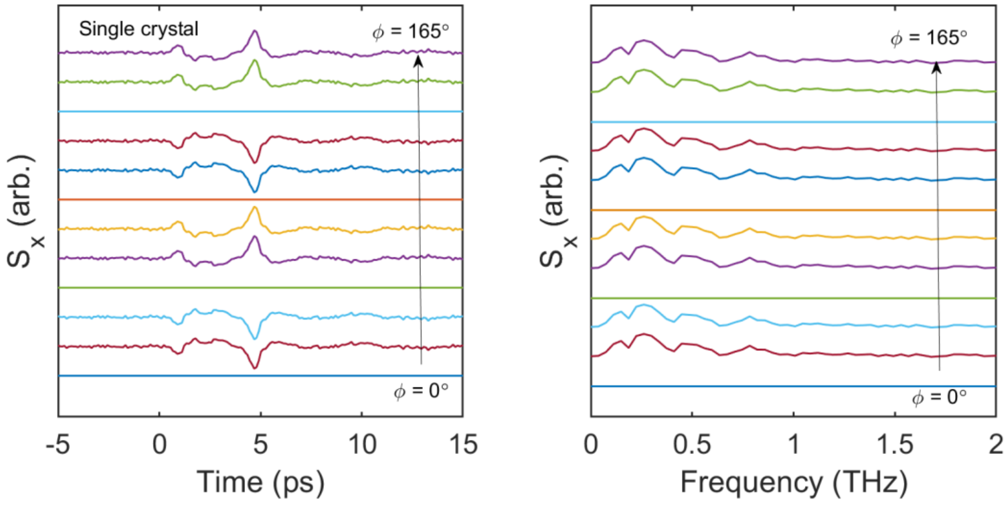

Figure S9. **a,** Emitted THz waveforms for the single crystal under illumination from different polarisation angles, ϕ. **b,** Corresponding FFT spectrum for **a.**

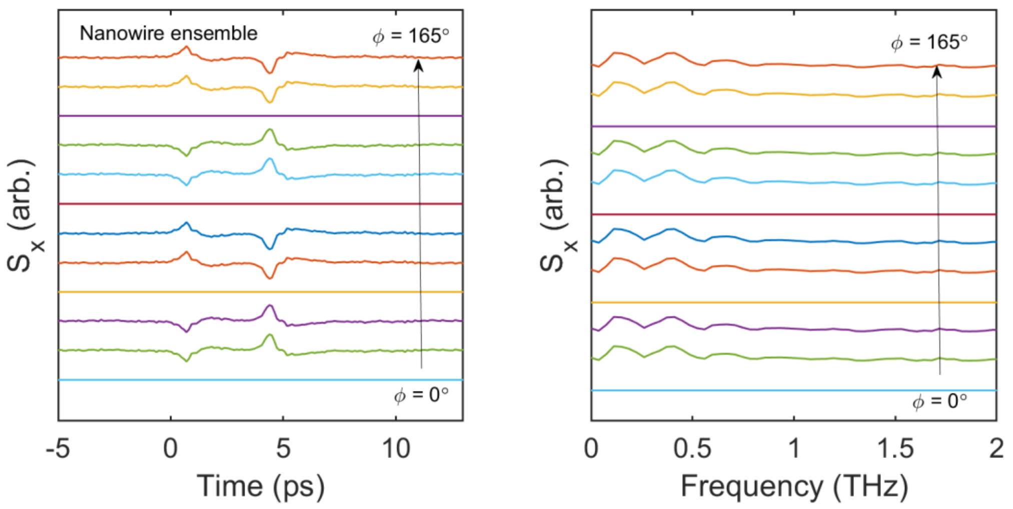

Figure S10. **a,** Emitted THz waveforms for the nanowire ensemble under illumination from different polarisation angles, ϕ. **b,** Corresponding FFT spectrum for **a.**

Figure S9 and S10 show the extracted $L_{1x}(t)$ emission contribution from the signal crystal and nanowire ensemble respectively for various degrees of polarisation. At normal incidence, there is negligible THz emission for illumination with both linear and circular polarisation. However, at elliptical polarisations, a clear signal is observed. For elliptically polarised photoexcitation, there is a component of the electric field in the direction normal to the surface, which allows LPGE photocurrents to contribute to the THz emission.

10. Linear polarisation dependence of THz emission

Figure S11 and S12 depict the dependence of the emitted THz pulse on linear polarisation angle for both the bulk single crystal and nanowire ensemble, respectively. A half wave plate was used to vary the angle of linear polarisation of the optical pump pulse. The emitted THz waveforms for each polarisation angle are shown in Figure S11a and S12a. For both samples, a clear $\cos 2\phi$ dependence was observed, as expected for emission due to bulk photothermal currents and shift currents.

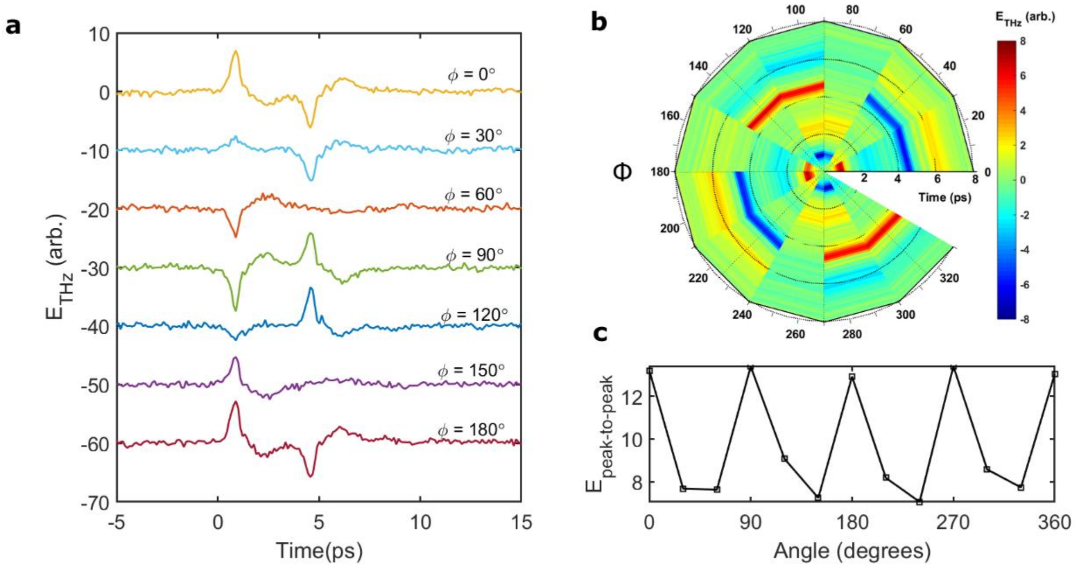

Figure S11 a, Emitted THz waveforms for the single crystal under illumination from different linear polarisation angles, ϕ. A halfwave plate is used to vary the angle of linear polarisation of the optical pump pulse. **b,** Polar plots of THz waveforms as a function of polarisation angle, ϕ, taken at α =180° (when THz emission is at a maximum in Figure 1e). The time delay is plotted along the radius and the polarisation angle against the circumference. The colour bar represents the amplitude of the THz emission. **c,** Peak-to-peak value of the emitted THz amplitude as a function of polarisation angle.

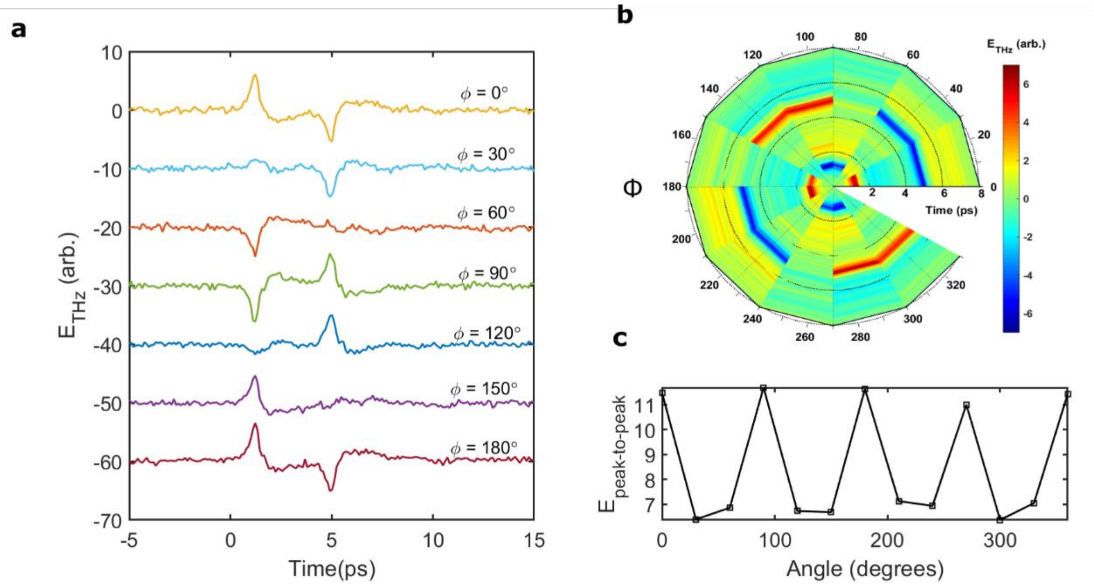

Figure S12 a, Emitted THz waveforms for the nanowire ensemble under illumination from different linear polarisation angles, ϕ. A halfwave plate is used to vary the angle of linear polarisation of the optical pump pulse. **b,** Polar plots of THz waveforms as a function of polarisation angle, ϕ, taken at α

=180° (when THz emission is at a maximum in Figure 2e). The time delay is plotted along the radius and the polarisation angle against the circumference. The colour bar represents the amplitude of the THz emission. **c,** Peak-to-peak value of the emitted THz amplitude as a function of polarisation angle.

11. Comparison of multi-cycle and few-cycle terahertz pulses

Figure S13 shows a direct comparison of the emitted THz pulses from the nanowire ensemble when photoexcited at the same crystal orientation at normal incidence with linear polarisation (polarised perpendicular to nanowire axis), which produces few-cycle pulses, and oblique incidence (45 degrees) with circular polarisation, which produces multi-cycle pulses. The amplitude of the multi-cycle emitted spectrum is 3.5 times larger than the amplitude of the few-cycle emitted spectrum. For a direct comparison, we have maintained the same crystal orientation at $\alpha = 90°$ to minimise contribution from surface optical rectification and bulk thermoelectric effects. However, we note that the amplitude of the few-cycle spectrum emitted under photoexcitation at normal incidence could be increased by changing the sample orientation, so that the photoexcitation light is polarised along the nanowire axis.

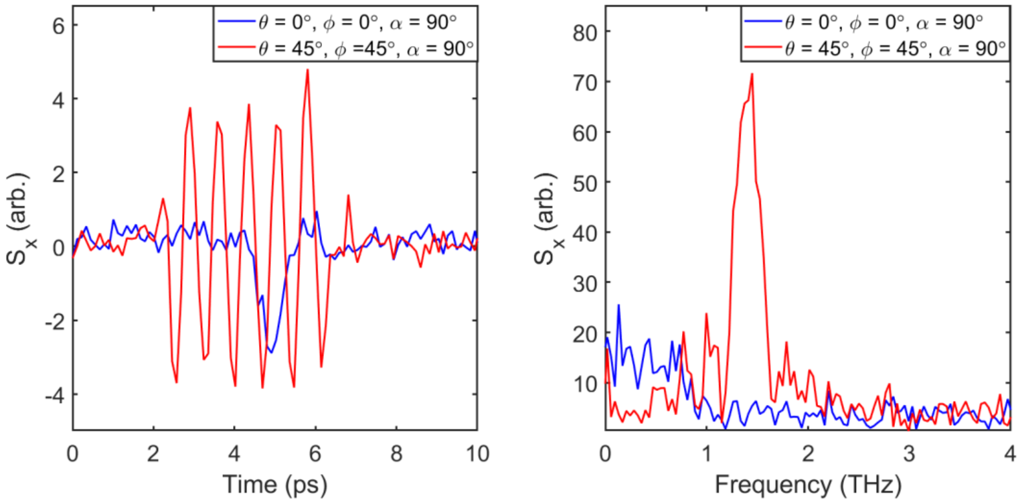

Figure S13. **a,** Time-domain trace of emitted THz pulse for Cd_3As_2 nanowire ensemble oriented with azimuthal angle, $\alpha = 90°$ when photoexcited at normal incidence with linear polarisation (blue) and at oblique incident angle of 45 degrees with circular polarisation (red) **b,** Corresponding FFT amplitude of time-domain traces in **a**.

12. Comparison of THz emission from ZnTe and Cd_3As_2 nanowires

Figure S134 shows a comparison of the THz emission from a 1 mm ZnTe crystal and Cd_3As_2 nanowire ensemble. Both samples were measured in the same experimental configuration with NIR photons with linear polarisation and the same excitation fluence. The THz emission from the nanowire ensemble is an order of magnitude smaller than the ZnTe crystal (5% of the measured ZnTe signal). However, we note that the nanowire ensemble has a much smaller material volume (4 order of magnitudes), highlighting the promise of this materials for on-chip THz source applications.

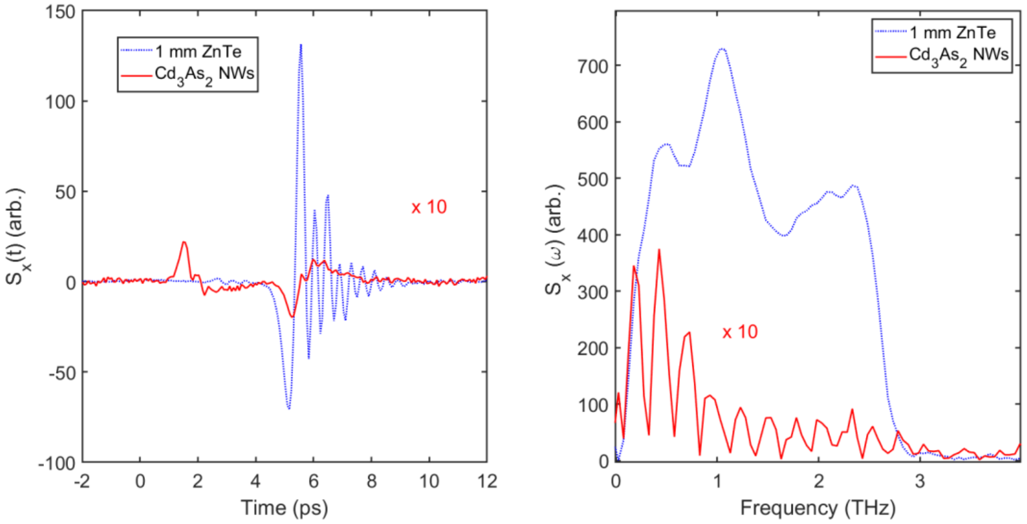

Figure S134. Time-domain trace of emitted THz pulse (left) and corresponding FFT amplitude spectrum (right) for a 1 mm ZnTe crystal (blue) and the Cd_3As_2 nanowire ensemble (red) when photoexcited at normal incidence with linear polarisation.

References

1. Liu, Z. K. *et al.* A stable three-dimensional topological Dirac semimetal Cd_3As_2. *Nat. Mater.* **13**, 677–681 (2014).
2. Schönherr, P. & Hesjedal, T. Structural properties and growth mechanism of Cd_3As_2 nanowires. *Appl. Phys. Lett.* **106**, 013115 (2015).
3. Neupane, M. *et al.* Observation of a three-dimensional topological dirac semimetal phase in high-mobility Cd_3As_2. *Nat. Commun.* **5**, 3786 (2014).
4. Cabellos, J. L. Second-order Optical Response in Semiconductors. *Phys. Rev. B* **61**, 5337- 5351 (2000).
5. Lewis, R. A. A review of terahertz sources. *J. Phys. D. Appl. Phys.* **47**, 374001 (2014).
6. Johnston, M. B., Whittaker, D. M., Corchia, A., Davies, A. G. & Linfield, E. H. Simulation of terahertz generation at semiconductor surfaces. *Phys. Rev. B - Condens. Matter Mater. Phys.* **65**, 1–8 (2002).
7. Klatt, G. *et al.* Terahertz emission from lateral photo-Dember currents. *Opt. Express* **18**, 4939–4947 (2010).
